# Study of In Silico Binding Interactions and In Vitro Biosorption of Type A Trichothecenes Using Devil Fish Chitosan

**DOI:** 10.3390/toxins18060263

**Published:** 2026-06-10

**Authors:** Martha Elena Aguilera Morales, Olga Nelly Rodríguez-Peña, Luis Barbo Hernández-Portilla, Cesar Mateo Flores-Ortíz

**Affiliations:** 1 Laboratorio Nacional en Salud, Facultad de Estudios Superiores Iztacala, Universidad Nacional Autónoma de México, Av. de los Barrios No. 1, Tlalnepantla 54090, Mexico; meaguilera@unpa.edu.mx (M.E.A.M.); lbarbo@unam.mx (L.B.H.-P.); 2 Laboratorio de Biogeoquímica, Unidad de Biología, Tecnología y Prototipos (UBIPRO), Facultad de Estudios Superiores Iztacala, Universidad Nacional Autónoma de México, Av. de los Barrios No. 1, Tlalnepantla 54090, Mexico; 3 Laboratorio de Fisiología Vegetal, Unidad de Biología, Tecnología y Prototipos (UBIPRO), Facultad de Estudios Superiores Iztacala, Universidad Nacional Autónoma de México, Av. de los Barrios No. 1, Tlalnepantla 54090, Mexico

**Keywords:** diacetoxyscirpenol (DAS), HT-2 toxin, mycotoxin binder, natural sequestrant, neosolaniol (NEO), T-2 toxin

## Abstract

Trichothecenes are the most common *Fusarium* mycotoxin contaminants of grains and their related products. Searching for effective adsorbents remains a major challenge in mycotoxicology, due to the low polarity and bulky chemical structure of type A trichothecenes. This study aimed to investigate in silico chitosan binding to type A trichothecenes such as diacetoxyscirpenol (DAS), neosolaniol (NEO), T-2 toxin (T2), and HT-2 toxin (HT2) and to study in vitro the devil fish chitosan biosorption capacity under two pH conditions (pHs 3 and 8). Molecular dynamic experiments showed that the chitosan monomers D-glucosamine and N-acetyl-D-glucosamine mostly bound to trichothecenes through the O in hydroxyls and glycosidic bonds and through their functional groups containing nitrogen. DAS exhibited a 9.44-, 6.39-, and 4.54-fold increase in the number of intermolecular contacts with chitosan compared to NEO, HT2 and T2, respectively. Moreover, in vitro experiments showed that at pH 3, chitosan exhibited a significant DAS sorption efficiency of 31.60% (*p* < 0.005), corresponding to a mass-normalized sorption capacity of 126.4 ng/mg. In contrast, no significant differences in sorption were observed at pH 8 (*p* > 0.05). Regarding NEO, T2, and HT2, no significant adsorption was detected under either pH condition (*p* > 0.05). This study is the first attempt to elucidate chitosan’s capacity to bind DAS and propose a mechanism for that interaction.

## 1. Introduction

Mycotoxins are low-molecular-weight secondary metabolites naturally produced by filamentous fungi [1]. They are known for being chemically heterogeneous and toxic compounds, capable of causing disease and even death in vertebrates, including humans [2]. Among the most important mycotoxins found contaminating seeds and grains are aflatoxins, ochratoxins, fumonisins, zearalenone and trichothecenes, which are produced by the genera Alternaria, Fusarium, Penicillium and Aspergillus [3]. Many countries have established maximum tolerable limits (MTLs) to prevent health damage due to mycotoxicosis [4], and it remains of scientific importance to develop more efficient alternatives for their control. Trichothecenes are the most common Fusarium mycotoxins found contaminating grains and related products such as cereals, beverages and animal food and feed [5,6]. They consist of non-volatile sesquiterpenes, whose general structure is a 12,13-epoxitrichothec-9-ene (EPT) three-ring molecule. According to their EPT substitution pattern, they are classified into four groups (Types A, B, C and D) [7]. Type A trichothecenes are characterized by the absence of a keto group at carbon eight (C8), containing instead an ester group, a hydroxyl group or no substituent [6,7], and include diacetoxyscirpenol (DAS), neosolaniol (NEO), T-2 toxin (T2) and HT-2 toxin (HT2) [8]. Both animals and humans can be exposed to simultaneous contamination by several trichothecenes; this is because Fusarium fungi are capable of co-producing these toxins, and a varied diet is generally composed of several grains and raw materials that may be contaminated [9]. The most common methods for detoxifying trichothecenes include the use of both inorganic and organic adsorbents. These materials act by binding to the mycotoxins (adsorbates), thereby trapping and inactivating them [10,11,12,13]. Both binder traits (chemical composition, pKa, structural configuration, and amount) and mycotoxin traits (size, shape and polarity), as well as environmental and experimental conditions (contact time, pH, and temperature), can affect mycotoxin adsorption and the mechanism of interaction [14,15,16,17,18].

Chitosan is a natural polyaminosacharide molecule derived from chitin. It is found in renewable sources such as fungi cell walls, crustacean shells, arthropod exoskeletons, and fish scales [19,20,21,22,23]. Its low toxicity, biocompatibility, and biodegradability and its antifungal, antibacterial, antioxidant and antitumoral properties, among other features, have led to its use in a wide variety of medical and biotechnological applications [24,25,26,27]. The chemical structure of chitosan consists of a linear polysaccharide chain of repeating units of D-glucosamine and N-acetyl-D-glucosamine linked by β–(1→4)-glycosidic bonds [28,29,30]. These monomers are naturally deacetylated to varying degrees depending on the source and other factors [31,32]. Given its chemical properties, chitosan has been used as a biosorbent for contaminants, such as heavy metals, owing to its chelating ability and magnetic separation [33,34]. Due to its abundant hydroxyl groups (OH) and nitrogen-containing functional groups (amines in D-glucosamines and amides in N-acetyl-D-glucosamines) that act as binding sites, chitosan has been used as biosorbent for dyes [35] and mycotoxins such as aflatoxins [36,37,38,39,40,41,42], fumonisins [36,38], ochratoxins [36,37,38,40,43], zearalenone [36,37,38,39,44], type A trichothecenes like T2 [36,38,45] and HT2 [38], and type B trichothecenes such as deoxynivalenol [36,38]. However, since type A trichothecenes are less polar than other mycotoxins, which prevents their strong binding to the adsorbent material, the study of effective adsorbents for trichothecenes remains a major challenge.

Therefore, to further investigate the mechanisms by which type A trichothecenes interact with chitosan, this study aimed to explore the following in silico: (i) the functional groups in the chitosan residues that interact with DAS, NEO, T2, and HT2 and (ii) the type of bonds that are formed within the chitosan–type A trichothecene complexes. For this purpose, a chitosan model of repeated units of D-glucosamine and N-acetyl-D-glucosamine linked by β–(1→4)-glycosidic bonds (~50,000 Da) was generated, and its interaction with each studied trichothecene was analyzed through molecular dynamic (MD) simulations. Additionally, laboratory biosorption experiments were performed at pH 3 and pH 8 using 2 ppm of each trichothecene. This concentration is almost 40 times higher than the 0.05 ppm maximum total limits (MTLs) for T2 toxin set by the official Mexican standards (NOM-188-SSA1-2002 [46] and PROY-NOM-000-ZOO-1999) [47]; almost 2 times higher than the 1 ppm MTLs for T2 and DAS in the Compendium of Mycotoxin Standards of the Food and Agriculture Organization of the United Nations (FAO) [48]; and almost 10 times higher than the 0.2 ppm MTLs established by the European Union (European Commission, 2006/576/EC) [49].

## 2. Results

### 2.1. In Silico Chitosan–Type A Trichothecenes

#### 2.1.1. Dipole Moment of Type A Trichothecenes

Dipole moment values were 2.877 D for DAS, 2.774 D for NEO, 2.675 D for T2, and 3.759 D for HT2.

#### 2.1.2. Chitosan–DAS Binding

**Figure 1:**
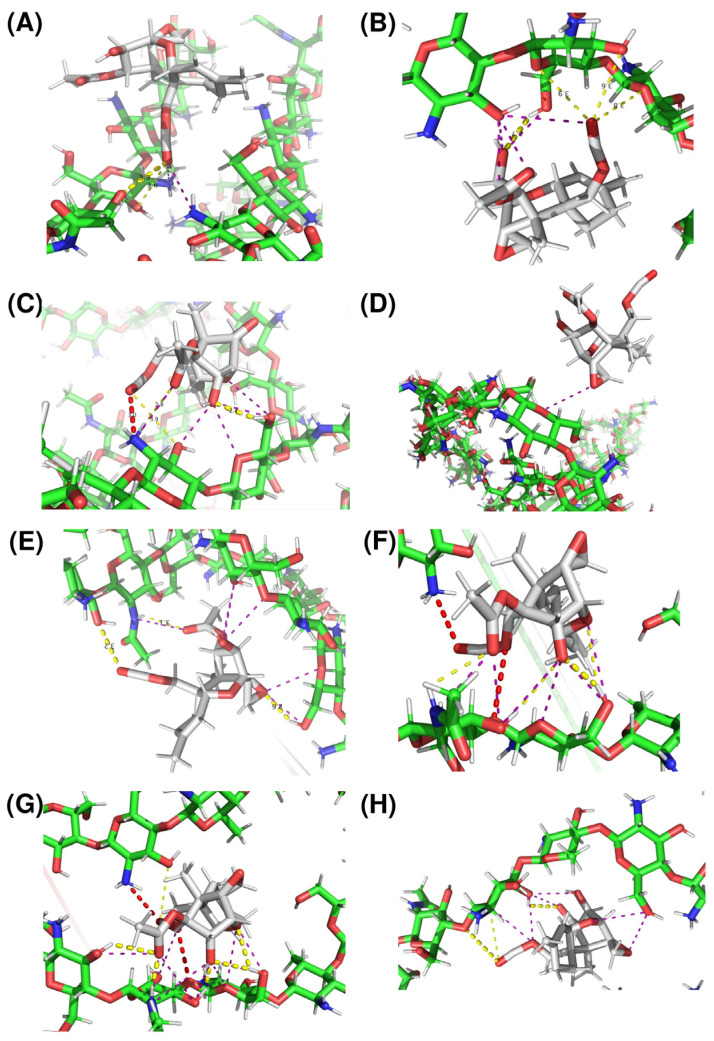
Representative contact points within the chitosan–DAS complex in molecular dynamics (100 ns). (**A**) DAS interacting with a hydroxyl O (C6) and the amine N of 2 D-glucosamines (0.2 ns); (**B**) DAS bound to 3 D-glucosamines through 8 bonds (1.0 ns); (**C**) DAS bound to a D-glucosamine by 9 bonds (60.2 ns); (**D**) DAS bound by its epoxide to the 1–5 glycosidic bond oxygen of D-glucosamine (78.2 ns); (**E**) DAS interacting with 4 D-glucosamines and with an N-acetylglucosamine (94.3 ns); (**F**) DAS bound to 2 D-glucosamines and an N-acetylglucosamine by 11 bonds (98.2 ns); (**G**) DAS bound through 19 bonds with two D-glucosamines and an N-acetylglucosamine (99.1 ns); (**H**) DAS bound through 9 bonds with two D-glucosamines (100 ns). Chitosan 80% deacetylated = green; DAS (diacetoxyscirpenol) = gray; polar bond = dashed red; non-polar bond = dashed purple; hydrogen bond = dashed yellow.

DAS bound at several points along the chitosan chain from 0.2 ns, throughout the entire simulation (100 ns). Chitosan and DAS showed 144 contact poses linked by 1 to 19 bonds (99.1 ns; Table S1) and interacted with up to 4 D-glucosamine and N-acetylglucosamine molecules at a time (94.3 ns). In some cases, they remained attached for more than 1 ns. DAS interacted more with D-glucosamine (70.19%) than with N-acetylglucosamine (20.68%) and 1,4-glycosidic bonds between a D-glucosamine and a N-acetylglucosamine (9.12%). Interactions were established through hydrogen bonding (24.10%), non-polar interactions (40.39%), hydrogen–non-polar interactions (26.38%), and polar bonds (9.12%; Table 1). D-glucosamine bound to DAS via the amine N, hydroxyl O at C3 and C6, and the glycosidic 1,4 and 1,5 glycosidic oxygens. N-acetylglucosamine bound through its amide carbonyl O, the amide N, the 1,4 and 1,5 glycosidic oxygens, and the hydroxyl O at C3 and C6. DAS bound through the ester carbonyl O at C4 and C15, the hydroxyl O at C3, the ether O of the ester at C4 and C15, the epoxide O, and the 2,11-ether oxygen (Figure 1). The DAS acetyl group at C4 participated in 18.07% of interactions, and its hydrogen at C8 did not interact with chitosan.

#### 2.1.3. Chitosan–NEO Binding

**Figure 2:**
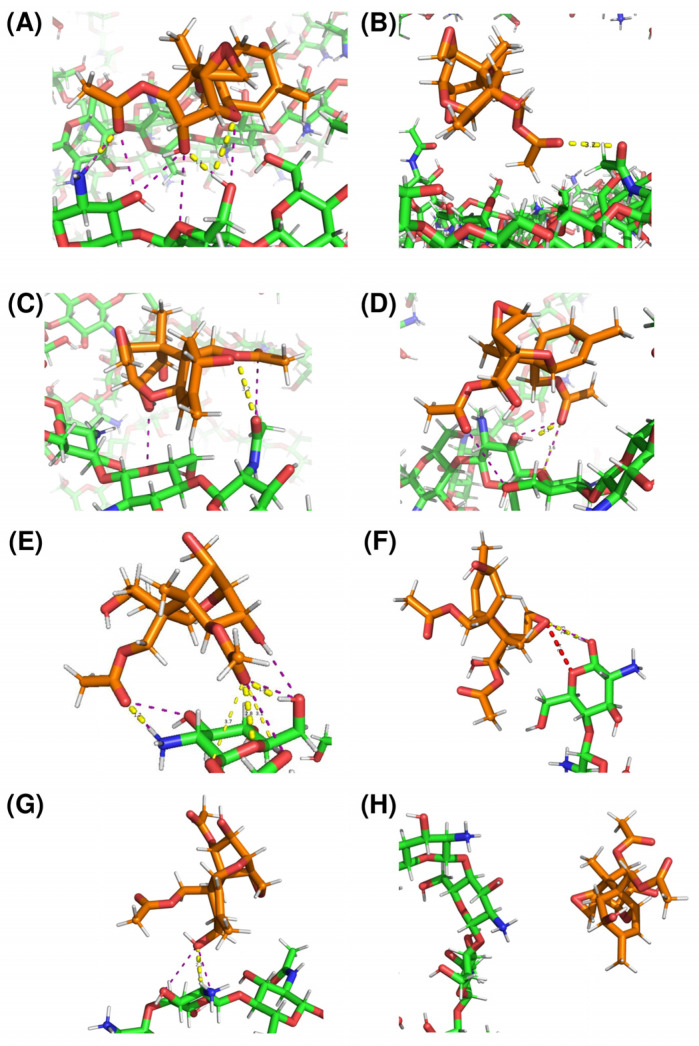
Representative contact points within the chitosan–NEO complex in molecular dynamics (100 ns). (**A**) NEO bound to two D-glucosamines by 9 bonds (0.2 ns); (**B**) NEO bound to the amide carbonyl O of an N-acetylglucosamine (0.6 ns); (**C**) NEO bound to a D-glucosamine and an N-acetylglucosamine (1.0 ns); (**D**) NEO bound to two D-glucosamines (1.1 ns); (**E**) NEO bound to a D-glucosamine by 10 bonds (47.9 ns); (**F**) NEO bound by its epoxide O through a hydrogen bond, a polar bond and a non-polar bond (58.2 ns); (**G**) NEO bound by its hydroxyl O (C8) to a D-glucosamine hydroxyl O (C3) and to the amine N (76.1 ns); (**H**) chitosan–NEO complex is broken (76.2 ns). Chitosan 80% deacetylated = green; NEO (neosolaniol) = orange; polar bond = dashed red; non-polar bond = dashed purple; hydrogen bond = dashed yellow.

NEO bound at several points on the chitosan chain from 0.2 ns until 76.1 ns was reached; subsequently, the complex dissociated, and NEO was no longer bound to chitosan (100 ns). Chitosan and NEO showed 26 contact poses, linked by 1 to 10 bonds (47.9 ns; Table S2), in which NEO interacted with D-glucosamine (95.38%) more than with N-acetylglucosamine (4.61%). Interactions were established through hydrogen bonding (9.23%), non-polar interactions (49.23%), and hydrogen–non-polar (40%) and polar bonds (1.53%; Table 1). NEO bound with up to 2 molecules of D-glucosamine at 23.07% of the contact points. D-glucosamine was linked through the hydroxyl O at C1, C3, C4 and C6, the amine N, and the 1,4 and 1,5 glycosidic bonds. N-acetylglucosamine bound through its amide-carbonyl O. NEO interacted via the ester carbonyl O at C4 and C15, the hydroxyl O in C3 and C8, the ether O of the ester in C4 and C15, the epoxide O and, the 2,11 glycosidic bond O (Figure 2). NEO bound through the glycosidic bond 2,11, hydroxyl O at C3, C8, ether O of the ester and the ester carbonyl O in C4 and C15 and the epoxide O. The acetyl group in NEO at C4 participated in 24.61% of the interactions, while the hydroxyl at C8 participated in 12.30%.

#### 2.1.4. Chitosan–T2 Binding

**Figure 3:**
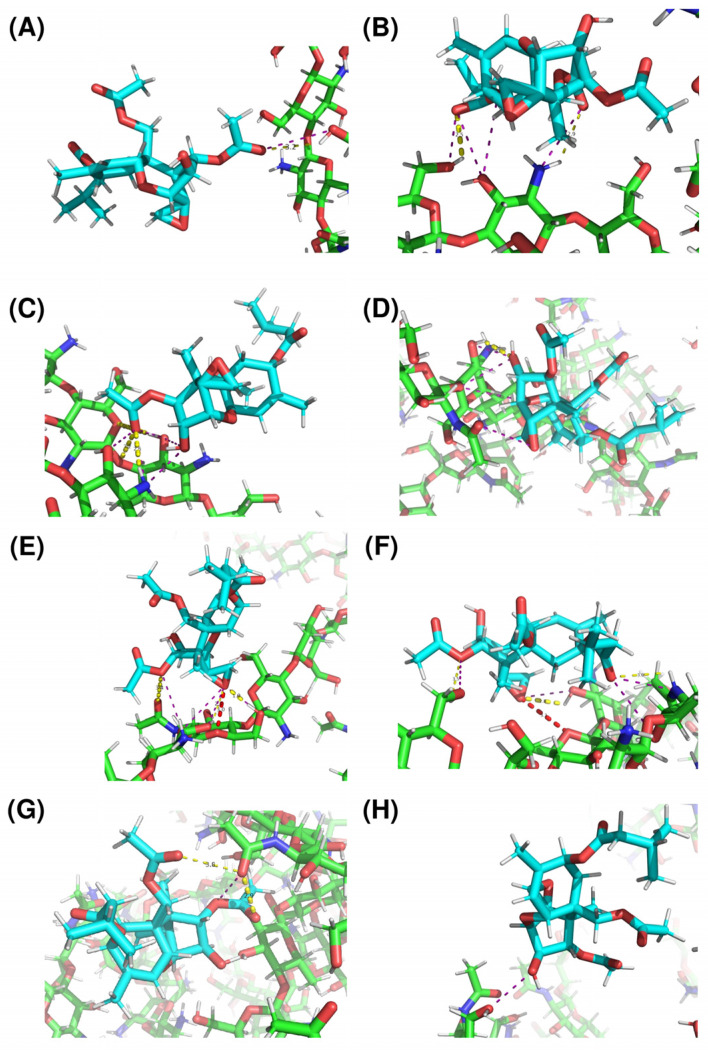
Representative contact points within the chitosan–T2 complex in molecular dynamics (100 ns). (**A**) T2 bound to a D-glucosamine (0.1 ns); (**B**) T2 bound to two D-glucosamines (1.1 ns); (**C**) T2 bound to three D-glucosamines (2.0 ns); (**D**) T2 bound to a D-glucosamine and an N-acetylglucosamine (2.9 ns); (**E**) T2 bound to a D-glucosamine and an N-acetylglucosamine (66.1–66.2 ns); (**F**) T2 bound to two D-glucosamines and an N-acetylglucosamine (85.3 ns); (**G**) T2 bound to an N-acetylglucosamine (99.5 ns); (**H**) T2 remains bound to an N-acetylglucosamine (100 ns). Chitosan 80% deacetylated = green; T2 (T2 toxin) = aqua; polar bond = dashed red; non-polar bond = dashed purple; hydrogen bond = dashed yellow.

T2 bound to several points along the chitosan chain, starting from 0.1 ns, and throughout the whole dynamic process (100 ns). Chitosan and T2 exhibited 50 contact poses, featuring between 1 and 11 bonds, and interacted with up to three D-glucosamine molecules simultaneously. In some cases, T2 remained attached for more than 1 ns (Table S3). T2 interacted more frequently with D-glucosamine (70.37%) than with N-acetylglucosamine (23.70%), while 5.92% of interactions involved the O 1,4 glycosidic bond between D-glucosamine and N-acetyl-D-glucosamine. Interactions consisted of hydrogen bonding (13.33%), non-polar interactions (51.85%), and hydrogen–non-polar (31.85%) and polar bonds (2.96%; Table 1). D-glucosamine bound to T2 via the hydroxyl oxygen atoms at C1, C3, C4 and C6, the amine nitrogen, and the 1,4-and 1,5-glycosidic bonds. N-acetylglucosamine joined through its amide nitrogen, amide carbonyl oxygen, and the hydroxyl oxygen at C3 and C6, as well as the 1,4- and 1,5-glycosidic bonds. T2 bound through the ester carbonyl oxygen at C4, C8 and C15; the hydroxyl oxygen at C3; the ether oxygen of the ester at C4, C8 and C15; the epoxide oxygen; and the 2,11 glycosidic bond oxygen (Figure 3). The acetyl group at C4 and the O-isovaleryl at C8 participated in 32.08% and 14.92% of the interactions, respectively.

#### 2.1.5. Chitosan–HT2 Binding

**Figure 4:**
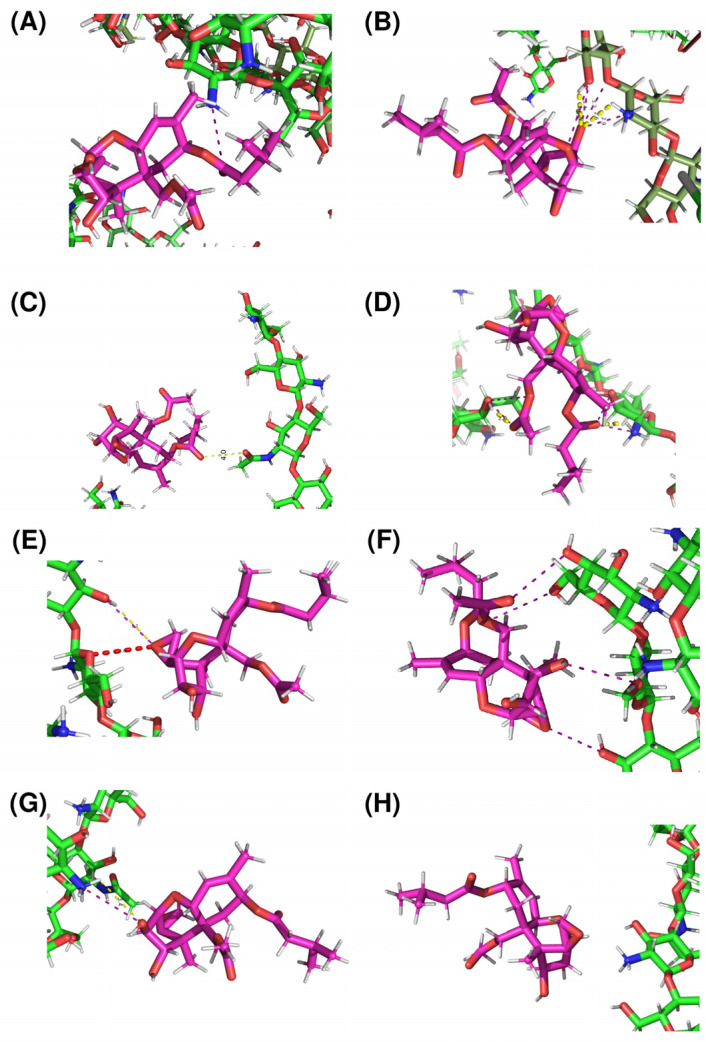
Representative contact points within the chitosan–HT2 complex during a 100 ns molecular dynamics (MD). (**A**) HT2 bound to a D-glucosamine (0.4 ns); (**B**) HT2 bound to a D-glucosamine (25.3 ns); (**C**) HT2 bound to an N-acetylglucosamine (40.2 ns); (**D**) HT2 bound to two D-glucosamines (60.2 ns); (**E**) HT2 bound to a D-glucosamine (65.5 ns); (**F**) HT2 bound to three D-glucosamines (66.9 ns); (**G**) HT2 bound to the amine nitrogen of a D-glucosamine (83.0 ns); (**H**) Dissociation of the chitosan–HT2 complex (83.1 ns). Chitosan 80% deacetylated = green; HT2 (HT2 toxin) = pink; polar bond = dashed red; non-polar bond = dashed purple; hydrogen bond = dashed yellow.

HT2 bound at several points on the chitosan chain throughout the dynamic process, from 0.4 ns until 83 ns, at which point the complex dissociated, and HT2 no longer bound to chitosan (100 ns). Chitosan and HT2 exhibited 47 contact poses with 1 to 7 bonds, interacting with up to three D-glucosamine molecules simultaneously. In some cases, HT2 remained attached for more than 1 ns (Table S4). HT2 interacted more frequently with D-glucosamine (95.83%) than with N-acetylglucosamine (4.16%). Interactions were mediated by hydrogen bonding (2.08%), non-polar interactions (54.16%), and hydrogen–non-polar (40.62%) and polar bonds (3.12%; Table 1). D-glucosamine bound to HT2 via the amine N; hydroxyl O at C3, C4 and C6; and the 1,4- 1,5-glycosidic bonds; N-acetylglucosamine joined through its amide carbonyl oxygen and the hydroxyl oxygen at C6. HT2 bound through the ester carbonyl oxygen at C4, C8 and C15; the hydroxyl oxygen at C3 and C4; the ether oxygen of the ester at C4, C8 and C15; the epoxide oxygen; and the 2,11 glycosidic bond oxygen (Figure 4). The HT2 acetyl at C4 participated in 47.91% of interactions, while the O-isovaleryl at C8 accounted for 3.13%.

### 2.2. In Vitro Experiments

#### 2.2.1. Chitosan Characterization

The chitosan exhibited a porosity of 65.19% and an apparent bulk density of 0.655 g/mL, indicating that it is a lightweight and highly porous material. Moreover, chitosan showed a water retention capacity of 19.77% and a high degree of deacetylation at 75.4%.

#### 2.2.2. Biosorption Assays

**Figure 5:**
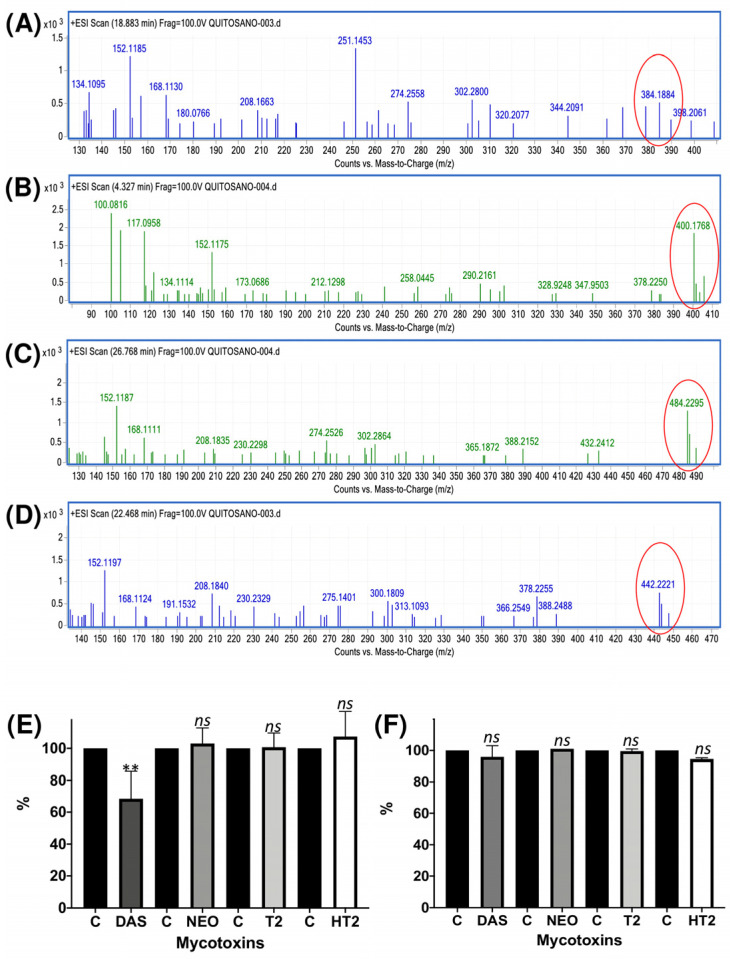
Biosorption of type A trichothecenes by chitosan. Characteristic ions shown by HPLC-ESI-TOF-MS for: (**A**) DAS; (**B**) NEO; (**C**) T2; (**D**) HT2; Red ellipses in panels (**A**–**D**) highlight the characteristic ions corresponding to each mycotoxin. Bars show the removal percentage of type A trichothecenes compared with their respective control (black); (**E**) biosorption experiments at pH 3; (**F**) biosorption experiments at pH 8. DAS = diacetoxyscirpenol; NEO = neosolaniol; T2 = T-2 toxin; and HT2 = HT-2 toxin. ** = *p* < 0.005; *ns* = non-significant.

When experiments were performed at pH 3, significant differences were found in the biosorption of the type A trichothecenes by chitosan (ANOVA; *p* = 0.0049; *F* = 4.712; DF = 7). Analyses of residual trichothecenes showed significant adsorption of DAS by chitosan (31.60%; (Sidak’s test *p* = 0.0038; t = 4.038; DF = 16; Figure 5) corresponding to a mass-normalized sorption capacity of 126.4 ng/mg. No differences were found for NEO, T2 and HT2. However, no significant differences were observed when experiments were performed at pH 8 (ANOVA; *p* = 0.07; *F* = 2.384; DF = 7).

## 3. Discussion

The physicochemical, mechanical, and biological traits of chitosan mainly depend on the distribution of acetyl groups, the degree of acetylation, and chain length [29,50]. Although several studies have tested chitosan–mycotoxin biosorption capacities using different mycotoxin types, to our knowledge, this is the first attempt to study chitosan as a trichothecene binder that considers both chitosan monomers (D-glucosamine and N-acetylglucosamine), includes DAS and NEO, and incorporates the use of chemoinformatic computational simulation tools. We found that (i) chitosan monomers mostly bound to type A trichothecenes through the oxygen in their hydroxyls and glycosidic bonds, as well as their functional groups containing nitrogen; (ii) D-glucosamine mostly bound through non-polar bonds to all the studied trichothecenes, whereas N-acetylglucosamine bound through hydrogen bonding to DAS, NEO, and HT2 and through non-polar bonds to T2; and (iii) DAS showed the highest binding frequency and biosorption percentage compared to the other studied trichothecenes. In this section, our most important findings and their implications are discussed.

Chitosan has advantages in food and feed safety contexts due to its ability to bind to common mycotoxins. The presence of abundant, reactive protonated amine (-NH^+3^) and hydroxyl (-OH) groups allows chitosan to undergo dynamic conformational adjustments. As shown by MDE simulations, this flexibility enables chitosan to structurally envelope non-planar structures, maximizing dipole–dipole and hydrogen bonding stabilization. Therefore, chitosan is a sustainable, targeted bioadsorbent capable of effectively overcoming the steric and chemical limitations of conventional mineral binders [51]. Yeast cell wall extracts, consisting mainly of β-glucans and glucomannans, offer other organic alternatives, yet their binding capacities are highly variable depending on the strain and extraction processing [52]. Chitosan may overcome this limitation by providing a highly reproducible chemical profile with customizable deacetylation degrees, ensuring stable and predictable adsorption [51]. In contrast, inorganic adsorbents, such as bentonites and modified clays, rely on rigid interlayer spaces and ion-exchange mechanisms which bind inefficiently with bulky trichothecenes due to structural mismatches and severe steric hindrance within the crystalline silicate sheets, unless they trap flat, highly polar molecules like aflatoxins [53]. Activated carbon provides non-specific porous trapping; however, its total lack of selectivity risks stripping essential nutrients, vitamins, and minerals from the matrix [54].

Results showed that both D-glucosamine and N-acetyl-D-glucosamine primarily bound to all the studied trichothecenes through the oxygen in hydroxyls and glycosidic bonds and the nitrogen found in the D-glucosamine amine and N-acetyl-D-glucosamine amide groups. Both hydroxyl (OH) and N-containing functional groups in chitosan have already been recognized for their capacity to bind adsorbates [55,56]. Hydroxyl group density in polymers has been found to contribute to the formation of a network that traps molecules, leading to improved adsorption capacity [55]. Due to its polarity and chemical composition (consisting of O^−^ and H^+^), the hydroxyl group (OH) can easily bind to other molecules. In this study, hydroxyls were found to bind via hydrogen bonds (HB), hydrogen–non-polar (HB; NP) and non-polar (NP) interactions. Non-polar interactions, also known as London dispersion forces, are considered the weakest interaction types since they constitute temporal intermolecular attractions [57]. Hydrogen bonding occurs when a H atom binds to an electronegative atom such as F, N or O; therefore, hydrogen bonds are stronger than non-polar interactions [58]. Chitosan is thought to be the only natural polysaccharide with an amino group [21]. Here, we revealed that not only does the N found in the D-glucosamine amine group contribute to mycotoxin binding, but so do the O and N in the amide group of N-acetyl-D-glucosamine and their hydroxyl groups. Although these interactions sites of N-acetyl-D-glucosamine accounted for only 29.8% (DAS); 4.61% (NEO); 29.6% (T2); 4.16% (HT2) of total binding in this study, this difference in the number of interactions is mainly due to: (i) the lower presence of N-acetyl-D-glucosamine within the chain compared to D-glucosamine, since an 80:20 ratio was used, and (ii) the fact that, unlike the D-glucosamine amine group, the amide in N-acetyl-D-glucosamine is not protonated. To our knowledge, this is the first attempt to consider the binding contribution of N-acetyl-D-glucosamine in chitosan. In this study, both amines and amides were found to bind through hydrogen bonding, as well as polar and non-polar interactions. Dipole–dipole polar interactions are formed because the differences in electronegativity create molecular dipoles [59]; therefore, among the interaction types found in this study, hydrogen bonds are the strongest, and polar interactions are stronger than the non-polar ones. However, their frequency and combined strength make these sequestrants significant. Future investigations should consider charge–charge interactions within chitosan–mycotoxin complexes.

In this study, we found that all tested type A trichothecenes bound to chitosan through the oxygen in their functional groups. A previous in silico study regarding chitosan and aflatoxins reported that the highest negative charge was found on the oxygen atoms [60]. We observed that DAS showed the highest number of interactions, in addition to the greatest biosorption percentage compared to the other three studied trichothecenes. Although the chemical structure of type A trichothecenes is very similar, the selective biosorption of DAS over other type A trichothecenes is driven by a critical balance between electrostatic complementarity and low steric hindrance at the C8 position. MDE (100 ns) revealed that DAS establishes a highly cooperative dynamic binding network with the chitosan, initiating with 8 hydrogen bonds and dipole–dipole interactions at 1.0 ns, and ultimately peaking at 19 concurrent bonds at 99.1 ns (see Figure 1). Because DAS only features a hydrogen atom (-H) at C8, it lacks bulky spatial payloads, which minimizes its molecular volume and allows it to enter the binding site of the chitosan where its strong local dipole moment can stabilize the network. Conversely, the presence of a hydroxyl group (-OH) in NEO and a bulky *O*-isovaleryl chain (-OCOCH_2_CH(CH_3_)_2_) in T2 and HT2 at this C8 position induces severe steric hindrance. This spatial obstruction physically blocks these mycotoxins from entering at the binding pockets and approaching the ideal texturing distances required for stable anchoring, effectively collapsing their binding networks and overriding any intrinsic polarity advantages. To our knowledge, this is the first attempt to study the biosorption capacity of chitosan–DAS and NEO. In this study, non-significant biosorption for T2 and HT2 was found. Consistent with our findings, previous studies have reported a poor adsorption for T2 [36,45] or non-significant results for both T2 and HT2 [38].

Moreover, although the 31.6% removal efficiency of DAS by chitosan at pH 3 found in this study may represent a modest threshold for industrial or commercial scales, it is important to highlight that the experimental concentration used (2 ppm) is considerably higher than the MTLs worldwide. This suggests that chitosan could exhibit higher relative efficiency when encountering the lower, environmentally realistic mycotoxin levels found in naturally contaminated grains, thereby offering a viable strategy to mitigate DAS exposure in food and feed chains. This limited performance in chitosan is mostly explained by the lower polarity of type A trichothecenes and the restricted surface area of chitosan flakes. To maximize chitosan’s detoxification capacity, optimization strategies should be explored. These include derivatization to introduce specialized functional groups into the polymer matrix to enhance hydrophobic interactions [61]; cross-linking to modify the chemical nature of the matrix, allowing hydrophobic compounds to be better retained via Van der Waals forces or dispersive interactions [36]; nanoparticle formation to reduce the material to the nanometer scale, thereby increasing the specific surface area and the availability of exposed active binding sites [62]; pH tuning to modify the protonation state of chitosan, optimizing electrostatic interactions [63]; and the fabrication of composite materials by coupling chitosan with rigid or porous materials, such as bentonite and activated carbon, which increases binding site density, adds complementary adsorption mechanisms, and improves structural stability [17,44,45,64,65,66]. Regarding pH, results showed chitosan–DAS biosorption at pH 3, in contrast to experiments performed at pH 8. Theoretically, the amine groups in D-glucosamine are protonated up to pH 8.5; however, as the pH becomes more acidic, a greater number of H^+^ ions on the chitosan surface are able to react with mycotoxins, leading to a better adsorption rate. Our results align with previous studies that found the same pattern under different pH conditions [54,67].

## 4. Conclusions

Overall, this study is the first attempt to provide a detailed description of the binding poses between functional groups of chitosan monomers and the type A trichothecenes (DAS, NEO, T2, and HT2). A significant efficiency in adsorbing DAS was identified for the first time in both in silico and in vitro approaches; this could be further improved by exploring optimization strategies to enhance chitosan binding efficiency in future research. This study supports the hypothesis that shape, polarity and molecular size are critical factors directing adsorption.

## 5. Materials and Methods

### 5.1. In Silico Chitosan–Type A Trichothecenes Experiments

To understand how chitosan interacts with type A trichothecenes, an assembly of multiple linear polysaccharide chains was modeled (80% deacetylated comprising 240 residues of poly [β–(1→4)–2–amino–2–deoxy–D–glucopyranose] (D-glucosamine) and 30 residues of 2-acetamido-2-deoxy-D-glucopyranose (N-acetylglucosamine), resulting in a total of 270 residues and a molecular weight of ~50,000 Da). The protonation states of D-glucosamine and N-acetylglucosamine were determined using MolGpKa (https://xundrug.cn/molgpka; accessed on 5 September 2025) [68]. Since the amino group in D-glucosamine remains protonated up to pH 8.3, its protonated form was used. For N-acetylglucosamine, the functional groups are deprotonated above pH 11.9; thus, its neutral form was considered. In brief, a chain of 27 residues of the sequence [1 N-acetylglucosamine-b(1→4)-(D-glucosamine)8]3 was modeled using the Glycan Reader and Modeler module of CHARMM-GUI (https://www.charmm-gui.org/; accessed on 7 September 2025). A total of ten chains were stacked into two blocks to achieve the 50,000 Da model using PyMOL (version 3.1.3.1) [69]. SMILES codes for the type A trichothecenes (DAS, NEO, T2, and HT2) were obtained from PubChem (https://pubchem.ncbi.nlm.nih.gov/; accessed on 7 September 2025; Table 2), drawn with ChemSketch (version 2021.2.0; www.acdlabs.com; accessed on 7 September 2025), and geometrically optimized (ground state, semi-empirical AM1 method, default spin) using Gaussian 09 (Revision D.01; Gaussian, Inc., Wallingford, CT, USA) [70]. The dipole moments of the trichothecenes were calculated in Debyes (D) using Avogadro (version 1.2.0) [71]. The topology files of the trichothecene models were obtained using the Ligand Reader and Modeler module of CHARMM-GUI. Each trichothecene was manually centered between the two chain blocks using PyMOL. Each chitosan–trichothecene complex was prepared using the PDB Reader and Manipulator module of CHARMM-GUI. The chitosan–trichothecene complexes in an aqueous solvent environment were prepared using the Solution Builder module in CHARMM-GUI [72]. A water box was constructed with a minimum distance of 10 Å from the chitosan edge to the box boundaries. The system was explicitly solvated using the TIP3P water model. The CHARMM36m force field was used [73], and the system was neutralized and adjusted to an ionic strength of 0.15 using K_2_HPO_4_ ions. Langevin dynamics was employed to maintain a temperature of 314.15 K (41 °C). The minimization process was set to 5000 steps (emtol = 1000 kJ/mol/nm). The equilibration process (canonical ensemble, NVT) was set to 5,000,000 steps (5 ns; 1.0 fs/step), and the final production run (isothermal–isobaric ensemble, NPT) was performed for 50,000,000 steps (100 ns; 0.2 fs/step). The molecular dynamic simulations were performed using GROMACS 2023.3 [74,75]. PyMOL was used to visualize the trajectories and create figures. Hydrogen-bond, polar, and non-polar interactions along the trajectories were identified using the Show contacts PyMOL plugin [76].

### 5.2. In Vitro Experiments

#### 5.2.1. Chitosan Characterization

The porosity of the chitosan extract was determined following the procedure described by Gonçalves et al., 2025 [77]. Briefly, a known mass of dry chitosan sample was weighed, submerged in a fixed volume of absolute ethanol, and kept at room temperature for 1h to ensure complete penetration into the polymer pores. The mixture containing the submerged sample was then weighed. Subsequently, the unabsorbed supernatant ethanol was decanted, and the wet chitosan mass recorded. Finally, a fresh volume of ethanol was reintroduced to the decanted sample container to determine the total combined weight and the internal porosity calculated. The apparent bulk density was determined using a Ray-Ran bulk density apparatus. In brief, a 100 mL volume of the dry granulated chitosan was gently poured into the standard measuring cylinder until completely filled without compaction. The mass of the contained sample was recorded using an analytical balance. The apparent bulk density was mathematically calculated through the direct mass-to-volume ratio. The degree of acetylation was determined by Fourier-transform infrared spectroscopy (FTIR). In brief, FTIR spectra were recorded using a diamond ATR accessory within the wavenumber range of 4000–650 cm^−1^. From the resulting spectrum, the absorbances at 1645 cm^−1^ and 3456 cm^−1^ were measured, corresponding to the amide group band (used to determine the N-acetyl group) and the hydroxyl group band, respectively. The degree of acetylation was subsequently calculated [78,79].

#### 5.2.2. Biosorption Assays

To investigate the sorption capacity of chitosan for type A trichothecenes and assess complex stability during simulated gastrointestinal transitions, in vitro biosorption assays were performed. In brief, a single solution was prepared containing chitosan extract from devil fish exoskeleton (0.5%), K_2_HPO_4_ ions (0.15 M), and 2 ppm of each type A trichothecene (DAS, NEO, T2, and HT2). To simulate the gastric-to-intestinal pH shift, assays were conducted at pH 3 and pH 8. Tubes were incubated at 40 ± 1 °C, for 24 h (replicating the gastrointestinal temperature of poultry), and centrifuged at 14,000 rpm for 3 min. The supernatant (850 µL) was recovered in 1.5 mL vials, evaporated to dryness under a nitrogen stream, and resuspended in 700 µL of HPLC-grade methanol (MeOH). All assays were conducted in triplicate, and data are presented as the removal percentage (%) of type A trichothecenes.

#### 5.2.3. Residual Trichothecenes Quantification

Quantification of trichothecenes was performed by reverse phase HPLC–ESI–TOF–MS, using positive ion mode, in an Agilent 1260 Infinity HPLC system (Agilent Technologies, Yishun, Singapore) with a RRHD Eclipse Plus C-18 column (1.8 μm, 2.1 × 100 mm; Agilent Technologies, Santa Clara, CA, USA); at 25 °C. The mobile phase consisted of (A) ammonium acetate (10 mM) and (B) HPLC-grade acetonitrile. The gradient was 80% A and 20% B. The flow rate was 0.15 mL/min, and the sample injection volume was 20 μL. The HPLC was coupled to an Agilent 66230B TOF/MS with an electrospray interface (Agilent Technologies, Santa Clara, CA, USA). The gas temperature was 350 °C, gas flow 6 L/min, nebulizer pressure 50 psig, fragmentor 105 V, skimmer 60 V, Oct RF 750 V, and capillary voltage 4000 V. The final precursor ions were identified as DAS = 384.2 *m*/*z*; NEO = 400.19 *m*/*z*; T2 = 484.25 *m*/*z*; and HT2 = 442.24 *m*/*z*. The scanning range was from 100 to 1000 *m*/*z*. Samples were injected in triplicate, and results were analyzed using Mass Hunter Data Acquisition software for 6200 series (version 5.01.5125; Agilent Technologies, USA) and Qualitative Analysis (version 6.0.633.10, Agilent Technologies, 2017).

#### 5.2.4. Data Analysis

Differences in the chitosan sorption capacity for DAS, NEO, T2 and HT2 toxins were determined by one-way multiple analysis of variance (ANOVA) followed by Sidak’s post hoc tests [80]; *p* < 0.05 was considered significant. Analyses were performed and figures were generated using GraphPad Prism 8^®^ (Version 8.4.0 for Mac OS).

## Figures and Tables

**Table 1:** Molecular interactions in chitosan–type A trichothecene complexes.

Molecular Interactions									
Type A Trichothecenes	Chitosan								
	D-glucosamine				N-acetylglucosamine				
	HB	P	NP	HB; NP	HB	P	NP	HB; NP	Total
DAS	85	37	193	116	63	19	55	46	614
NEO	4	1	31	26	3	-	-	-	65
T2	8	3	50	34	10	1	20	9	135
HT2	-	3	51	38	2	-	1	1	96

DAS = diacetoxyscirpenol; NEO = neosolaniol; T2 = T-2 toxin; HT2 = HT-2 toxin. HB = hydrogen bond; P = polar interaction; NP = non-polar interaction; HB; NP = hydrogen bond and non-polar interaction.

**Table 2:** Chemical structures of type A trichothecenes.

ID	IUPAC Name	Molecular Weight (g/mol)	Two-Dimensional Structure
DAS	[(1S,2R,7R,9R,10R,11S,12S)-11-acetyloxy-10-hydroxy-1,5-dimethylspiro[8-oxatricyclo[7.2.1.02,7]dodec-5-ene-12,2′-oxirane]-2-yl]methyl acetate	366.4	** 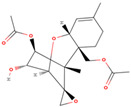 **
NEO	[(1S,2R,4S,7R,9R,10R,11S,12S)-11-acetyloxy-4,10-dihydroxy-1,5-dimethylspiro[8-oxatricyclo[7.2.1.02,7]dodec-5-ene-12,2′-oxirane]-2-yl]methyl acetate	382.4	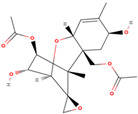
T2	[(1S,2R,4S,7R,9R,10R,11S,12S)-11-acetyloxy-2-(acetyloxymethyl)-10-hydroxy-1,5-dimethylspiro[8-oxatricyclo[7.2.1.02,7]dodec-5-ene-12,2′-oxirane]-4-yl] 3-methylbutanoate	466.5	** 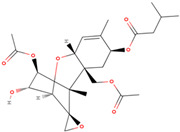 **
HT2	[(1S,2R,4S,7R,9R,10R,11S,12S)-2-(acetyloxymethyl)-10,11-dihydroxy-1,5-dimethylspiro[8-oxatricyclo[7.2.1.02,7]dodec-5-ene-12,2′-oxirane]-4-yl] 3-methylbutanoate	424.5	** 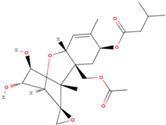 **

Diacetoxyscirpenol (DAS), neosolaniol (NEO), T-2 toxin (T2) and HT-2 toxin (HT2). IUPAC names were calculated using Lexichem TK 2.7.0 from PubChem. The chemical structures of type A trichothecenes are very similar. Variations are present at C4, top left side (DAS, NEO and T2 toxin have an acetyl (OAc) group, while HT2 has a hydroxyl (OH) instead) and C8, top right side (DAS has a hydrogen (H); NEO has a hydroxyl (OH), and T2 and HT2 have an O-isovaleryl (OCOCH_2_CH(CH_3_)_2_) group).

## Data Availability

The original contributions presented in this study are included in the article/Supplementary Material. Further inquiries can be directed to the corresponding authors.

## Supplementary Materials

The following supporting information can be downloaded at: https://www.mdpi.com/article/10.3390/toxins18060263/s1, Table S1: Chitosan–diacetoxyscirpenol (DAS) binding poses; Table S2: Chitosan–neosolaniol (NEO) binding poses; Table S3: Chitosan–T2 toxin (T2) binding poses; Table S4: Chitosan–HT2 toxin (HT2) binding poses.

## Abbreviations

The following abbreviations are used in this manuscript:

DAS	Diacetoxyscirpenol
NEO	Neosolaniol
T2	T-2 toxin
HT2	HT-2 toxin
HB	Hydrogen bond
P	Polar interaction
NP	Non-polar interaction
HB; NP	Hydrogen bond and non-polar interaction
MTLs	Maximum Tolerable Limits
