# Supplementary material for: Study of In Silico Binding Interactions and In Vitro Biosorption of Type A Trichothecenes Using Devil Fish Chitosan
Source: Toxins (Basel). 2026 Jun 10;18(6):263. doi: 10.3390/toxins18060263 (PMC13308271; doi:10.3390/toxins18060263)
Supplement: Supplementary file 1 [file toxins-18-00263-s001.zip › Table S1. Chitosan-diacetoxyscirpenol (DAS) binding poses.pdf]

Table S1. Contact points and interaction types between chitosan former molecules and the type A trichothecene Diacetoxyscirpenol (DAS).

Chitosan-DAS interactions					
Binding Sites					
Contact Pose	Time (ns)	Interaction type	Chitosan		DAS
			D-glucosamine	N-acetylglucosamine	
1	0.2	hb	hydroxyl O (C6) (1)		ester carbonyl O (C15)
		hb	amine N (2)		ester carbonyl O (C15)
		np	amine N (2)		ester carbonyl O (C15)
2	0.3	hb	hydroxyl O (C3) (1)		ester carbonyl O (C15)
		p	amine N (1)		ester carbonyl O (C15)
3	0.4	hb	hydroxyl O (C6) (1)		ester carbonyl O (C15)
		np	hydroxyl O (C3) (2)		ester carbonyl O (C15)
		p	amine N (2)		ester carbonyl O (C15)
4	0.5	np	hydroxyl O (C6) (1)		ester carbonyl O (C15)
		hb	glycosidic bond O 1-5 (1)		ester carbonyl O (C15)
		np	glycosidic bond O 1-4 (1-2)		ester carbonyl O (C15)
		np	hydroxyl O (C3) (2)		ester carbonyl O (C15)
5	0.6	hb; np	amine N (1)		ester carbonyl O (C15)
		p	amine N (1)		ester carbonyl O (C15)
6	0.7	np	amine N (1)		ester carbonyl O (C15)
7	0.8	np	glycosidic bond O 1-5 (1)		ether O of the ester (C15)
		hb	glycosidic bond O 1-5 (1)		ester carbonyl O (C15)
		hb	hydroxyl O (C3) (2)		ester carbonyl O (C15)
		np	amine N (2)		ester carbonyl O (C15)
		np	hydroxyl O (C6) (2)		glycosidic bond O 2-11
		hb; np	hydroxyl O (C6) (2)		hydroxyl O (C3)
		hb; np	glycosidic bond O 1-5 (2)		hydroxyl O (C3)
		hb	glycosidic bond O 1-4 (1-2)		ester carbonyl O (C4)
		np	hydroxyl O (C3) (2)		ester carbonyl O (C4)
8	0.9	np	hydroxyl O (C6) (1)		glycosidic bond O 2-11
		hb; np	hydroxyl O (C6) (1)		hydroxyl O (C3)
		hb; np	glycosidic bond O 1-5 (1)		hydroxyl O (C3)

		np<br>hb; np	hydroxyl O (C3) (1)<br>amine N (1)		hydroxyl O (C3)<br>ester carbonyl O (C4)
9	1.0	hb<br>hb<br>hb<br>np<br>hb; np<br>np<br>np	glycosidic bond O 1-5 (1)<br>hydroxyl O (C3) (2)<br>glycosidic bond O 1-5 (2)<br>hydroxyl O (C3) (3)<br>hydroxyl O (C6) (3)<br>hydroxyl O (C3) (3)<br>hydroxyl O (C3) (3)		ester carbonyl O (C15)<br>ester carbonyl O (C15)<br>ester carbonyl O (C15)<br>ester carbonyl O (C15)<br>hydroxyl O (C3)<br>ester carbonyl O (C4)<br>ether O of the ester (C4)
10	1.1	hb<br>hb; np	hydroxyl O (C6)<br>hydroxyl O (C6)		ester carbonyl O (C15)<br>ether O of the ester (C4)
11	1.2	hb<br>hb<br>np<br>np<br>np<br>hb; np	glycosidic bond O 1-5 (1)<br>hydroxyl O (C3) (2)<br>hydroxyl O (C3) (3)<br>hydroxyl O (C3) (3)<br>hydroxyl O (C3)<br>amine (3)		ester carbonyl O (C15)<br>ether O of the ester (C15)<br>ether O of the ester (C15)<br>hydroxyl O (C3)<br>ester carbonyl O (C4)<br>ester carbonyl O (C4)
12	1.3	hb<br>hb<br>np<br>hb; np<br>hb; np	glycosidic bond O 1-5 (1)<br>hydroxyl O (C3) (2)<br>hydroxyl O (C6) (2)<br>hydroxyl O (C3) (3)<br>amine N (3)		ester carbonyl O (C15)<br>ester carbonyl O (C15)<br>ether O of the ester (C15)<br>ester carbonyl O (C4)<br>ester carbonyl O (C4)
13	1.4	np<br>np<br>hb; np<br>hb; np<br>np<br>hb<br>hb<br>np<br>np<br>np	hydroxyl O (C6) (1)<br>hydroxyl O (C6) (1)<br>hydroxyl O (C6) (2)<br>hydroxyl O (C6) (2)<br>glycosidic bond O 1-5 (2)<br>glycosidic bond O 1-5 (2)<br>glycosidic bond O 1-4 (2-3)<br>hydroxyl O (C3) (3)<br>hydroxyl O (C3) (3)<br>hydroxyl O (C3) (3)		ester carbonyl O (C15)<br>ether O of the ester (C15)<br>glycosidic bond O 2-11<br>hydroxyl O (C3)<br>hydroxyl O (C3)<br>ester carbonyl O (C4)<br>ester carbonyl O (C4)<br>ester carbonyl O (C4)<br>ether O of the ester (C4)<br>hydroxyl O (C3)
14	1.5	hb; np<br>hb	hydroxyl O (C6) (1)<br>hydroxyl O (C3) (1)		hydroxyl O (C3)<br>ester carbonyl O (C15)

		hb; np<br>np	glycosidic bond O 1-4 (1-2)<br>glycosidic bond O 1-5 (2)		hydroxyl O (C3)<br>ester carbonyl O (C4)
15	1.6	hb<br>hb; np<br>hb; np<br>np<br>hb; np<br>hb; np<br>np<br>np<br>np	glycosidic bond O 1-4 (1-2)<br>amine N (2)<br>glycosidic bond O 1-4 (1-2)<br>glycosidic bond O 1-5 (2)<br>hydroxyl O (C6) (2)<br>hydroxyl O (C3) (2)<br>glycosidic bond O 1-5 (3)<br>hydroxyl O (C6) (3)<br>hydroxyl O (C6) (3)		ester carbonyl O (C4)<br>ester carbonyl O (C4)<br>hydroxyl O (C3)<br>hydroxyl O (C3)<br>hydroxyl O (C3)<br>ester carbonyl O (C15)<br>ether O of the ester (C15)<br>ester carbonyl O (C15)<br>ether O of the ester (C15)
16	1.7	np<br>hb<br>hb; np<br>hb; np<br>np<br>np	hydroxyl O (C3) (1)<br>glycosidic bond O 1-4 (1-2)<br>glycosidic bond O 1-5 (2)<br>hydroxyl O (C6) (2)<br>hydroxyl O (C6) (2)<br>hydroxyl O (C6) (3)		hydroxyl O (C3)<br>hydroxyl O (C3)<br>hydroxyl O (C3)<br>hydroxyl O (C3)<br>ether O of the ester (C15)<br>ester carbonyl O (C15)
17	1.8	hb; np	hydroxyl O (C6)		glycosidic bond O 2-11
18	2.4	p<br>hb	amine N<br>hydroxyl O (C3)		ester carbonyl O (C15)<br>ester carbonyl O (C15)
19	2.6	np<br>p	hydroxyl O (C3)<br>glycosidic bond O 1-5		hepoxide O<br>hepoxide O
20	12.8	np<br>np	hydroxyl O (C3) (1)<br>hydroxyl O (C6) (2)		hepoxide O<br>hepoxide O
21	12.9	hb; np	amine N		glycosidic bond O 2-11
22	13.1	np<br>p<br>hb; np	hydroxyl O (C3) (1)<br>glycosidic bond O 1-5 (2)<br>hydroxyl O (C6) (2)		hepoxide O<br>hepoxide O<br>hepoxide O
23	20.9	hb	hydroxyl O (C6)		ester carbonyl O (C15)
24	21.2	hb; np	hydroxyl O (C3)		hepoxide O
25	21.3	np	hydroxyl O (C6)		hepoxide O
26	21.4	np	glycosidic bond O 1-5		hepoxide O
27	21.5	np	glycosidic bond O 1-5		hydroxyl O (C3)
28	36.3	np	hydroxyl O (C6) (1)		ether O of the ester (C15)

		np<br>hb	hydroxyl O (C6) (1)<br>hydroxyl O (C3) (2)		ester carbonyl O (C15)<br>ester carbonyl O (C15)
29	36.5	np	hydroxyl O (C6)		hydroxyl O (C3)
30	36.6	np	hydroxyl O (C6)		ester carbonyl O (C4)
31	36.7	hb; np<br>hb	hydroxyl O (C6)<br>glycosidic bond O 1-4		ether O of the ester (C15)<br>ester carbonyl O (C4)
32	36.8	np<br>np	glycosidic bond O 1-5<br>glycosidic bond O 1-4		ether O of the ester (C15)<br>hydroxyl O (C3)
33	36.9	hb<br>hb<br>np	glycosidic bond O 1-5 (1)<br>glycosidic bond O 1-4 (1-2)<br>glycosidic bond O 1-4 (1-2)		ester carbonyl O (C15)<br>ester carbonyl O (C15)<br>hydroxyl O (C3)
34	37	hb<br>np<br>hb	glycosidic bond O 1-5 (1)<br>hydroxyl O (C6) (2)<br>glycosidic bond O 1-4 (1-2)		ester carbonyl O (C15)<br>ether O of the ester (C15)<br>ester carbonyl O (C4)
35	37.1	np<br>hb<br>np<br>hb; np<br>hb; np<br>hb; np	hydroxyl O (C6) (1)<br>hydroxyl O (C6) (2)<br>hydroxyl O (C6) (2)<br>hydroxyl O (C6) (2)<br>glycosidic bond O 1-5 (2)<br>hydroxyl O (C3) (3)		ester carbonyl O (C15)<br>ester carbonyl O (C15)<br>ether O of the ester (C15)<br>hydroxyl O (C3)<br>hydroxyl O (C3)<br>hydroxyl O (C3)
36	37.3	hb<br>hb; np<br>hb; np<br>np<br>np<br>hb; np	glycosidic bond O 1-4 (1-2)<br>glycosidic bond O 1-5 (1)<br>hydroxyl O (C6) (1)<br>hydroxyl O (C6) (2)<br>hydroxyl O (C6) (2)<br>hydroxyl O (C6) (2)		ester carbonyl O (C15)<br>ether O of the ester (C15)<br>ether O of the ester (C15)<br>ether O of the ester (C15)<br>hydroxyl O (C3)<br>ester carbonyl O (C4)
37	37.4	hb<br>np<br>hb; np<br>np<br>hb; np	glycosidic bond O 1-4 (1-2)<br>glycosidic bond O 1-5 (1)<br>hydroxyl O (C6) (1)<br>hydroxyl O (C6) (2)<br>hydroxyl O (C6) (2)		ester carbonyl O (C15)<br>ether O of the ester (C15)<br>ether O of the ester (C15)<br>ether O of the ester (C15)<br>hydroxyl O (C3)
38	37.5	np<br>hb; np	hydroxyl O (C6)<br>hydroxyl O (C6)		hydroxyl O (C3)<br>ester carbonyl O (C4)
39	37.7	np	hydroxyl O (C3)		hepoxide O

40	39.2	hb	hydroxyl O (C6)		ester carbonyl O (C15)
41	59.2	hb<br>p	glycosidic bond O 1-4<br>amine N		ester carbonyl O (C15)<br>ester carbonyl O (C15)
42	59.3	np	hydroxyl O (C6)		hydroxyl O (C3)
43	59.7	hb		amide carbonyl O	ester carbonyl O (C15)
44	59.9	hb		amide carbonyl O	hepoxide O
45	60	hb; np	amine N		ester carbonyl O (C4)
46	60.1	hb; np<br>np<br>np<br>np	amine N<br>hydroxyl O (C3)<br>hydroxyl O (C3)<br>glycosidic bond O 1-5		ester carbonyl O (C4)<br>ester carbonyl O (C4)<br>hydroxyl O (C3)<br>hydroxyl O (C3)
47	60.2	p<br>hb; np<br>hb<br>np<br>np<br>hb; np<br>np	amine N (1)<br>amine N (1)<br>hydroxyl O (C3) (1)<br>hydroxyl O (C3) (1)<br>glycosidic bond O 1-5 (2)<br>hydroxyl O (C6) (2)<br>hydroxyl O (C6) (2)		ester carbonyl O (C15)<br>ester carbonyl O (C4)<br>ester carbonyl O (C15)<br>hydroxyl O (C3)<br>hydroxyl O (C3)<br>hydroxyl O (C3)<br>glycosidic bond O 2-11
48	60.3	hb; np	hydroxyl O (C6)		glycosidic bond O 2-11
49	60.4	hb; np<br>p<br>hb	hydroxyl O (C6) (1)<br>amine (2)<br>hydroxyl O (C3) (2)		hydroxyl O (C3)<br>ester carbonyl O (C15)<br>ester carbonyl O (C15)
50	60.5	hb; np<br>hb; np	amine N<br>amine N		hydroxyl O (C3)<br>glycosidic bond O 2-11
51	60.9	np<br>np<br>hb; np	hydroxyl O (C6)<br>hydroxyl O (C6)<br>hydroxyl O (C6)		ether O of the ester (C15)<br>hydroxyl O (C3)<br>ester carbonyl O (C4)
52	61	np	hydroxyl O (C6)		ether O of the ester (C15)
53	61.1	np<br>hb<br>np<br>np<br>hb	glycosidic bond O 1-4<br>glycosidic bond O 1-4 	 <br>amide N<br>amide N<br>amide carbonyl O	ether O of the ester (C15)<br>ester carbonyl O (C15)<br>ester carbonyl O (C15)<br>ether O of the ester (C15)<br>ether O of the ester (C15)
54	61.2	np	glycosidic bond O 1-4 (1-2)		ether O of the ester (C15)

		hb	glycosidic bond O 1-4 (12)		ester carbonyl O (C15)
		p		amide N	ester carbonyl O (C15)
		hb		amide carbonyl O	ester carbonyl O (C15)
		hb		amide carbonyl O	hydroxyl O (C3)
55	61.3	np	hydroxyl O (C6)		ether O of the ester (C15)
		hb	hydroxyl O (C6)		ester carbonyl O (C15)
56	61.6	np	hydroxyl O (C6)		hepoxide O
57	62.4	p	amine N		ester carbonyl O (C15)
		hb	glycosidic bond O 1-4		ester carbonyl O (C15)
		hb	hydroxyl O (C6)		ester carbonyl O (C15)
58	62.5	np	hydroxyl O (C6)		hydroxyl O (C3)
59	63	np	hydroxyl O (C3)		ester carbonyl O (C4)
60	63.2	np	hydroxyl O (C6)		ester carbonyl O (C4)
		hb		hydroxyl O (C3)	ester carbonyl O (C15)
		hb		acetyl carbonyl O	ester carbonyl O (C15)
		np		acetyl carbonyl O	ether O of the ester (C15)
61	63.3	hb	glycosidic bond O 1-5 (1)		ester carbonyl O (C15)
		hb	glycosidic bond O 1-4		ester carbonyl O (C15)
		np		hydroxyl O (C3)	ester carbonyl O (C15)
62	63.4	np	amine N		ester carbonyl O (C15)
		np	hydroxyl O (C6)		ester carbonyl O (C4)
		np	hydroxyl O (C3)		ester carbonyl O (C4)
63	63.5	p	amine N		ester carbonyl O (C15)
64	63.6	p	amine N		ester carbonyl O (C15)
		np	hydroxyl O (C3)		hydroxyl O (C3)
		np		amide N	hydroxyl O (C3)
65	63.7	np	glycosidic bond O 1-5		hydroxyl O (C3)
		np	hydroxyl O (C6)		glycosidic bond O 2-11
66	63.8	np	amine N		ester carbonyl O (C15)
		hb; np	amine N(another)		hydroxyl O (C3)
67	63.9	np	glycosidic bond O 1-5 (1)		hydroxyl O (C3)
		hb; np	hydroxyl O (C3) (2)		hydroxyl O (C3)
		np	glycosidic bond O 1-5 (2)		glycosidic bond O 2-11
		np	glycosidic bond O 1-4 (2)		glycosidic bond O 2-11

		hb; np	hydroxyl O (C3)		glycosidic bond O 2-11
68	78.2	np	glycosidic bond O 1-5		hepoxide O
69	78.3	np<br>np	hydroxyl O (C6)<br>glycosidic bond O 1-5		hepoxide O<br>hepoxide O
70	83.4	hb	glycosidic bond O 1-5		ester carbonyl O (C15)
71	83.5	np<br>hb; np<br>hb; np<br>np	glycosidic bond O 1-5<br>hydroxyl O (C6)<br>hydroxyl O (C6)<br>hydroxyl O (C6)		ester carbonyl O (C15)<br>ester carbonyl O (C15)<br>hydroxyl O (C3)<br>ester carbonyl O (C4)
72	83.6	np<br>hb; np	hydroxyl O (C3)<br>amine N		hepoxide O<br>hepoxide O
73	83.7	hb; np	amine N		hepoxide O
74	85.6	hb<br>hb<br>np	glycosidic bond O 1-5<br>glycosidic bond O 1-4<br>hydroxyl O (C3)		ester carbonyl O (C15)<br>ester carbonyl O (C15)<br>ester carbonyl O (C15)
75	91	hb; np<br>np	amine N<br>hydroxyl O (C6)		hydroxyl O (C3)<br>ester carbonyl O (C4)
76	91.1	np<br>np	hydroxyl O (C6)<br>hydroxyl O (C6)		ether O of the ester (C15)<br>ester carbonyl O (C15)
77	91.2	np<br>np<br>hb; np<br>np<br>np	glycosidic bond O 1-5<br>hydroxyl O (C6)<br>hydroxyl O (C6)<br>glycosidic bond O 1-5<br>hydroxyl O (C3)		ester carbonyl O (C4)<br>ester carbonyl O (C4)<br>hydroxyl O (C3)<br>hydroxyl O (C3)<br>hydroxyl O (C3)
78	91.3	np<br>p<br>np	hydroxyl O (C3)<br>amine N<br>amine N		ester carbonyl O (C15)<br>ester carbonyl O (C15)<br>ether O of the ester (C15)
79	91.4	hb; np<br>hb; np<br>np	hydroxyl O (C3)<br>amine N<br>hydroxyl O (C6)		ether O of the ester (C15)<br>ether O of the ester (C15)<br>ester carbonyl O (C4)
80	91.5	hb; np<br>hb; np<br>np<br>hb	amine N<br>amine N<br>glycosidic bond O 1-5<br>hydroxyl O (C6)		hydroxyl O (C3)<br>ester carbonyl O (C4)<br>ether O of the ester (C15)<br>ester carbonyl O (C15)

		np	hydroxyl O (C6)		ether O of the ester (C15)
81	91.6	np	hydroxyl O (C6)		ether O of the ester (C15)
		hb	hydroxyl O (C6)		ester carbonyl O (C15)
82	91.7	hb; np	hydroxyl O (C6) (1)		ether O of the ester (C15)
		np	hydroxyl O (C6) (1)		ester carbonyl O (C15)
		hb	glycosidic bond O 1-5 (1)		ester carbonyl O (C15)
		np		hydroxyl O (C3)	ester carbonyl O (C15)
		hb; np		glycosidic bond O 1-5	hydroxyl O (C3)
		hb; np	hydroxyl O (C3) (2)		hydroxyl O (C3)
		np		amide N	hydroxyl O (C3)
		hb; np		amide N	ester carbonyl O (C4)
		hb	glycosidic bond O 1-4 (2)		ester carbonyl O (C4)
83	91.8	hb		hydroxyl O (C3)	ester carbonyl O (C15)
		np		hydroxyl O (C3)	ether O of the ester (C15)
		hb; np		hydroxyl O (C6)	hydroxyl O (C3)
		hb; np		glycosidic bond O 1-5	hydroxyl O (C3)
		hb; np		amide N	ester carbonyl O (C4)
		np	glycosidic bond O 1-4		hydroxyl O (C3)
		np	hydroxyl O (C3)		hydroxyl O (C3)
84	91.9	hb; np		amide N	hydroxyl O (C3)
		hb; np		amide N	ester carbonyl O (C4)
		hb	glycosidic bond O 1-4		hydroxyl O (C3)
85	92	hb	hydroxyl O (C6) (1)		ester carbonyl O (C15)
		hb	glycosidic bond O 1-5 (1)		ester carbonyl O (C15)
		hb		hydroxyl O (C6)	ester carbonyl O (C15)
		hb		glycosidic bond O 1-5	ester carbonyl O (C15)
		hb		hydroxyl O (C6)	ester carbonyl O (C15)
		np	hydroxyl O (C3) (2)		ether O of the ester (C15)
		hb; np		amide N	ester carbonyl O (C4)
		hb	glycosidic bond O 1-4 2		ester carbonyl O (C4)
86	92.1	np	hydroxyl O (C6)		ester carbonyl O (C15)

		hb		glycosidic bond O 1-5	ether O of the ester (C15)
		p		glycosidic bond O 1-4	ether O of the ester (C15)
		hb; np		amide N	ester carbonyl O (C4)
87	92.2	hb	glycosidic bond O 1-4 (1-2)		ester carbonyl O (C15)
		np	glycosidic bond O 1-4 (1-2)		ether O of the ester (C15)
		np	glycosidic bond O 1-5 (2)		hydroxyl O (C3)
		np	hydroxyl O (C6) (3)		hydroxyl O (C3)
88	92.3	np	hydroxyl O (C6)		hydroxyl O (C3)
89	92.4	np	hydroxyl O (C6)		hydroxyl O (C3)
		np	hydroxyl O (C6)		ester carbonyl O (C4)
90	92.5	np	hydroxyl O (C6) (1)		hydroxyl O (C3)
		hb; np	hydroxyl O (C6) (2)		ester carbonyl O (C4)
		np	hydroxyl O (C6) (3)		ether O of the ester (C4)
91	93.1	hb; np	hydroxyl O (C6) (1)		hepoxide O
		np	amine N (2)		ester carbonyl O (C4)
92	93.4	hb	hydroxyl O (C3)		ester carbonyl O (C4)
93	93.9	np	hydroxyl O (C3)		hepoxide O
		hb		amide carbonyl O	ether O of the ester (C4)
94	94.1-94.2	np	glycosidic bond O 1-5		hepoxide O
95	94.3	hb; np	hydroxyl O (C6) (1)		hepoxide O
		np	glycosidic bond O 1-5 (1)		hepoxide O
		np	glycosidic bond O 1-4 (2-3)		hydroxyl O (C3)
		np	hydroxyl O (C3) (3)		hydroxyl O (C3)
		hb; np		amide N	ester carbonyl O (C4)
		hb	hydroxyl O (C3) (4)		ester carbonyl O (C15)
96	94.4	p	glycosidic bond O 1-5 (1)		hepoxide O
		np	hydroxyl O (C3) (2)		ether O of the ester (C4)
		np	hydroxyl O (C3) (2)		ester carbonyl O (C4)
		hb; np	amine N (3)		ester carbonyl O (C4)
		np		hydroxyl O (C3)	ester carbonyl O (C4)
		np		hydroxyl O (C3)	ether O of the ester (C4)
		np		hydroxyl O (C3)	ether O of the ester (C15)
		hb		amide carbonyl O	ether O of the ester (C15)

97	94.5	np	hydroxyl O (C3)		hydroxyl O (C3)
		np	amine N		hydroxyl O (C3)
		hb; np		amide N	ester carbonyl O (C4)
		np	hydroxyl O (C3)		ether O of the ester (C4)
		np	hydroxyl O (C3)		ester carbonyl O (C4)
		hb		amide carbonyl O	ether O of the ester (C15)
		hb		amide carbonyl O	ester carbonyl O (C15)
98	94.6	p	glycosidic bond O 1-5 (1)		hepoxide O
		hb	glycosidic bond O 1-4 (2)		ester carbonyl O (C4)
		hb	glycosidic bond O 1-5 (2)		ester carbonyl O (C4)
		hb; np		hydroxyl O (C3)	ester carbonyl O (C4)
		hb		hydroxyl O (C3)	ester carbonyl O (C15)
		np		hydroxyl O (C3)	ether O of the ester (C15)
		hb		amide carbonyl O	ester carbonyl O (C15)
99	94.7	p	glycosidic bond O 1-5 (1)		hepoxide O
		hb; np	hydroxyl O (C3) (2)		hydroxyl O (C3)
		hb	glycosidic bond O 1-5 (3)		ester carbonyl O (C4)
		np		hydroxyl O (C3)	ether O of the ester (C15)
		np	hydroxyl O (C6) (3)		ester carbonyl O (C4)
		np	hydroxyl O (C6) (3)		ether O of the ester (C15)
100	94.8	p	glycosidic bond O 1-5 (1)		hepoxide O
		hb		amide carbonyl O	ether O of the ester (C15)
		hb	hydroxyl O (C6) (2)		ester carbonyl O (C15)
		np		amide carbonyl O	ether O of the ester (C15)
		hb; np		hydroxyl O (C3)	ester carbonyl O (C15)
		hb	glycosidic bond O 1-5 (2)		ester carbonyl O (C4)
		hb; np	hydroxyl O (C3) (2)		ester carbonyl O (C4)
101	94.9-95.2	hb		amide carbonyl O	ether O of the ester (C15)
		hb	hydroxyl O (C6)		ester carbonyl O (C15)
		np		amide carbonyl O	ether O of the ester (C15)
		hb; np		hydroxyl O (C3)	ester carbonyl O (C15)
		hb	glycosidic bond O 1-5		ester carbonyl O (C4)
		hb; np	hydroxyl O (C3)		ester carbonyl O (C4)
102	95.3	p	glycosidic bond O 1-5 (1)		hepoxide O

		np	glycosidic bond O 1-4 (1-2)		hepoxide O
		hb; np		amide N	hydroxyl O (C3)
		np		hydroxyl O (C3)	hydroxyl O (C3)
		hb	glycosidic bond O 1-5 (3)		ester carbonyl O (C4)
		hb; np	hydroxyl O (C3) (3)		ester carbonyl O (C4)
103	95.4	hb; np	amine N		hepoxide O
		hb; np	hydroxyl O (C6)		ester carbonyl O (C4)
104	95.7	np	hydroxyl O (C3)		hydroxyl O (C3)
		np	hydroxyl O (C3)		ester carbonyl O (C4)
		hb; np	amine N		ester carbonyl O (C4)
105	96.1	np		hydroxyl O (C3)	ether O of the ester (C15)
		np		hydroxyl O (C3)	ester carbonyl O (C15)
		hb	glycosidic bond O 1-4		ester carbonyl O (C15)
		hb	glycosidic bond O 1-5		ester carbonyl O (C15)
		hb	hydroxyl O (C6)		ester carbonyl O (C15)
		hb	hydroxyl O (C6)		ether O of the ester (C15)
106	96.2	np	hydroxyl O (C3) (1)		hepoxide O
		np	glycosidic bond O 1-5 (2)		hepoxide O
		hb; np	hydroxyl O (C6) (2)		hepoxide O
		hb; np		amide N	hydroxyl O (C3)
		np		hydroxyl O (C3)	ether O of the ester (C15)
		np	glycosidic bond O 1-5 (3)		ether O of the ester (C15)
		hb	glycosidic bond O 1-4 (3)		ester carbonyl O (C15)
		hb	glycosidic bond O 1-5 (3)		ester carbonyl O (C15)
		hb	hydroxyl O (C6) (3)		ester carbonyl O (C15)
		np	hydroxyl O (C6) (3)		ether O of the ester (C15)
107	96.3	hb; np	hydroxyl O (C3) (1)		hepoxide O
		p	glycosidic bond O 1-5 (2)		hepoxide O
		np		hydroxyl O (C3)	ether O of the ester (C15)
		np		hydroxyl O (C3)	ester carbonyl O (C15)
		hb	glycosidic bond O 1-5 (3)		ester carbonyl O (C15)
		np	hydroxyl O (C6) (3)		ester carbonyl O (C15)
108	96.4	hb; np	hydroxyl O (C6)		ether O of the ester (C15)
109	96.5	np	hydroxyl O (C6)		ether O of the ester (C4)

110	96.6	np	amine N (1)			ether O of the ester (C4)
		np	hydroxyl O (C3) (1)			ether O of the ester (C15)
		np	glycosidic bond O 1-5 (2)			ester carbonyl O (C15)
111	96.7	np	glycosidic bond O 1-4 (1-2)			ether O of the ester (C15)
		np	glycosidic bond O 1-4 (1-2)			ester carbonyl O (C15)
		np	hydroxyl O (C3) (1)			ester carbonyl O (C15)
		hb	glycosidic bond O 1-5 (2)			ester carbonyl O (C15)
112	96.8	hb	hydroxyl O (C3) (1)			ester carbonyl O (C15)
		hb; np	hydroxyl O (C3) (1)			ester carbonyl O (C4)
		hb	glycosidic bond O 1-4 (1-2)			ester carbonyl O (C4)
113	96.9	p	amine N (1)			ester carbonyl O (C15)
		hb	hydroxyl O (C3) (1)			ester carbonyl O (C15)
114	97.0	hb; np	hydroxyl O (C6) (1)			glycosidic bond O 2-11
		hb; np	hydroxyl O (C6) (1)			hydroxyl O (C3)
		np	glycosidic bond O 1-5 (1)			hydroxyl O (C3)
		np		glycosidic bond O 1-4		ether O of the ester (C15)
		hb		glycosidic bond O 1-4		ester carbonyl O (C15)
		hb; np			hydroxyl O (C3)	ester carbonyl O (C4)
		hb			hydroxyl O (C6)	ester carbonyl O (C15)
115	97.1	hb; np	hydroxyl O (C3) (1)			hydroxyl O (C3)
		np	glycosidic bond O 1-5 (1)			hydroxyl O (C3)
		p		glycosidic bond O 1-4		ether O of the ester (C15)
		hb			hydroxyl O (C6)	ester carbonyl O (C15)
		hb; np			hydroxyl O (C3)	hydroxyl O (C3)
		hb; np			hydroxyl O (C3)	ester carbonyl O (C4)
		np			amide N	ester carbonyl O (C4)
		np	amine N (2)			ester carbonyl O (C15)
116	97.2	np	glycosidic bond O 1-5 (1)			hepoxide O
		np	hydroxyl O (C6) (1)			ether O of the ester (C15)
		hb	hydroxyl O (C6) (1)			ester carbonyl O (C15)
		hb	glycosidic bond O 1-5 (1)			ester carbonyl O (C15)
		hb		glycosidic bond O 1-4		ester carbonyl O (C15)
		np			hydroxyl O (C3)	ester carbonyl O (C15)
		np			hydroxyl O (C3)	ether O of the ester (C15)

117	97.3	hb; np	hydroxyl O (C6) (1)			ether O of the ester (C15)
		p	glycosidic bond O 1-5 (1)			ether O of the ester (C15)
		hb	glycosidic bond O 1-5 (1)			ester carbonyl O (C15)
		hb		glycosidic bond O 1-4		ester carbonyl O (C15)
		np			hydroxyl O (C3)	ether O of the ester (C15)
118	97.4	hb; np	hydroxyl O (C6) (1)			hydroxyl O (C3)
		np	glycosidic bond O 1-5 (1)			ether O of the ester (C15)
		hb		glycosidic bond O 1-4		ester carbonyl O (C15)
119	97.5	hb			hydroxyl O (C6)	ester carbonyl O (C15)
		hb		glycosidic bond O 1-4		ester carbonyl O (C15)
		hb; np	hydroxyl O (C6) (1)			hydroxyl O (C3)
120	97.6	hb	glycosidic bond O 1-4 (1-2)			ester carbonyl O (C15)
		np	glycosidic bond O 1-4 (1-2)			ether O of the ester (C15)
		np	glycosidic bond O 1-5 (1)			ether O of the ester (C15)
		hb; np	hydroxyl O (C6) (1)			hydroxyl O (C3)
121	97.7	hb			amide carbonyl O	ester carbonyl O (C4)
		np			hydroxyl O (C3)	ester carbonyl O (C4)
		p	amine N (1)			ester carbonyl O (C15)
		hb		glycosidic bond O 1-4 (2)		ester carbonyl O (C15)
		np			hydroxyl O (C3)	ether O of the ester (C15)
		np		glycosidic bond O 1-4 (2)		ether O of the ester (C15)
		np	amine N (2)			ester carbonyl O (C15)
		p	glycosidic bond O 1-5 (2)			ether O of the ester (C15)
		hb; np	hydroxyl O (C6) (2)			hydroxyl O (C3)
		hb; np	hydroxyl O (C6) (2)			glycosidic bond O 2-11
122	97.8	p	amine N (1)			ester carbonyl O (C15)
		hb	hydroxyl O (C3) (1)			ester carbonyl O (C15)
		hb		glycosidic bond O 1-4 (2)		ester carbonyl O (C15)
		p		glycosidic bond O 1-4 (2)		ether O of the ester (C15)
		np			hydroxyl O (C3)	ether O of the ester (C15)
		np	glycosidic bond O 1-5 (2)			ether O of the ester (C15)
		hb; np			amide N	ester carbonyl O (C4)
		np	glycosidic bond O 1-5 (2)			hydroxyl O (C3)
		np			hydroxyl O (C3)	ester carbonyl O (C4)

		hb; np	hydroxyl O (C6) (2)	hydroxyl O (C3)
		hb; np	hydroxyl O (C6) (2)	glycosidic bond O 2-11
		np	glycosidic bond O 1-5 (3)	hepoxide O
123	97.9	p	amine N (1)	ester carbonyl O (C15)
		hb	hydroxyl O (C3) (1)	ester carbonyl O (C15)
		hb	glycosidic bond O 1-4 (2)	ester carbonyl O (C15)
		np	hydroxyl O (C3)	ether O of the ester (C15)
		np	glycosidic bond O 1-5 (2)	ether O of the ester (C15)
		hb; np	amide N	ester carbonyl O (C4)
		np	glycosidic bond O 1-5 (2)	hydroxyl O (C3)
		np	hydroxyl O (C3)	ester carbonyl O (C4)
		hb; np	hydroxyl O (C6) (2)	hydroxyl O (C3)
		np	hydroxyl O (C6) (2)	glycosidic bond O 2-11
124	98.0	p	amine N (1)	ester carbonyl O (C15)
		hb; np	amide N	ester carbonyl O (C4)
		p	glycosidic bond O 1-4 (2)	ether O of the ester (C15)
		hb; np	hydroxyl O (C3)	hydroxyl O (C3)
		np	glycosidic bond O 1-5 (2)	hydroxyl O (C3)
		hb; np	hydroxyl O (C6) (2)	hydroxyl O (C3)
		hb; np	hydroxyl O (C6) (2)	glycosidic bond O 2-11
125	98.1	p	amine N (1)	ester carbonyl O (C15)
		hb	glycosidic bond O 1-4 (2)	ester carbonyl O (C15)
		hb; np	hydroxyl O (C3)	hydroxyl O (C3)
		np	glycosidic bond O 1-5 (2)	hydroxyl O (C3)
		hb; np	hydroxyl O (C6) (2)	hydroxyl O (C3)
		hb; np	hydroxyl O (C6) (2)	glycosidic bond O 2-11
126	98.2	p	amine N (1)	ester carbonyl O (C15)
		hb; np	amide N	ester carbonyl O (C4)
		p	glycosidic bond O 1-4 (2)	ether O of the ester (C15)
		hb; np	hydroxyl O (C3)	hydroxyl O (C3)
		np	glycosidic bond O 1-5 (2)	hydroxyl O (C3)
		hb; np	hydroxyl O (C6) (2)	hydroxyl O (C3)
		hb; np	hydroxyl O (C6) (2)	glycosidic bond O 2-11
127	98.3	p	amine N (1)	ester carbonyl O (C15)

		p		glycosidic bond O 1-4 (2)		ether O of the ester (C15)
		hb			hydroxyl O (C6)	ester carbonyl O (C15)
		np		glycosidic bond O 1-4 (2)		ester carbonyl O (C15)
		hb; np			amide N	ester carbonyl O (C4)
		np			hydroxyl O (C3)	ester carbonyl O (C4)
		np			hydroxyl O (C3)	hydroxyl O (C3)
		np	glycosidic bond O 1-5 (2)			hydroxyl O (C3)
		np	glycosidic bond O 1-5 (2)			ether O of the ester (C15)
		hb; np	hydroxyl O (C6) (2)			hydroxyl O (C3)
		np	hydroxyl O (C6) (2)			glycosidic bond O 2-11
128		np	glycosidic bond O 1-5 (1)			hepoxide O
		hb	hydroxyl O (C3) (2)			ester carbonyl O (C15)
		p	amine N (2)			ester carbonyl O (C15)
		np	amine N (3)			ester carbonyl O (C15)
		hb		glycosidic bond O 1-4 (3)		ester carbonyl O (C15)
		hb			hydroxyl O (C6)	ester carbonyl O (C15)
129	98.5	np	amine N			ester carbonyl O (C15)
		hb	hydroxyl O (C3) (1)			ester carbonyl O (C15)
		hb		glycosidic bond O 1-4 (2)		ester carbonyl O (C15)
		np	glycosidic bond O 1-5 (2)			ether O of the ester (C15)
		hb; np	hydroxyl O (C6) (2)			hydroxyl O (C3)
		np	hydroxyl O (C6) (2)			glycosidic bond O 2-11
130	98.6	np	hydroxyl O (C3) (1)			ester carbonyl O (C4)
		hb; np			amide N	ester carbonyl O (C4)
		hb; np			hydroxyl O (C3)	hydroxyl O (C3)
		np	glycosidic bond O 1-5 (2)			hydroxyl O (C3)
		hb; np	hydroxyl O (C6) (2)			hydroxyl O (C3)
		np	hydroxyl O (C6) (2)			glycosidic bond O 2-11
		p		glycosidic bond O 1-4 (2)		ether O of the ester (C15)
		hb		glycosidic bond O 1-4 (2)		ester carbonyl O (C15)
		np			hydroxyl O (C6)	ester carbonyl O (C15)
131	98.7	hb	hydroxyl O (C6) (1)			ester carbonyl O (C15)
		hb		glycosidic bond O 1-4 (2)		ester carbonyl O (C15)
		p		glycosidic bond O 1-4 (2)		ether O of the ester (C15)

		np	glycosidic bond O 1-5 (2)	hydroxyl O (C3)
		hb; np	hydroxyl O (C6) (2)	hydroxyl O (C3)
		hb; np	hydroxyl O (C6) (2)	glycosidic bond O 2-11
132	98.8	p	amine N (1)	ester carbonyl O (C15)
		np	hydroxyl O (C6)	ester carbonyl O (C15)
		hb	glycosidic bond O 1-4 (2)	ester carbonyl O (C15)
		p	glycosidic bond O 1-4 (2)	ether O of the ester (C15)
		hb; np	amide N	ester carbonyl O (C4)
		hb; np	hydroxyl O (C3)	hydroxyl O (C3)
		np	glycosidic bond O 1-5 (2)	hydroxyl O (C3)
		hb; np	hydroxyl O (C3) (2)	hydroxyl O (C3)
		hb; np	hydroxyl O (C6) (2)	glycosidic bond O 2-11
133	98.9	np	amine N (1)	ester carbonyl O (C15)
		hb	hydroxyl O (C3) (1)	ester carbonyl O (C15)
		np	hydroxyl O (C6)	ester carbonyl O (C15)
		hb	glycosidic bond O 1-4 (2)	ester carbonyl O (C15)
		p	glycosidic bond O 1-4 (2)	ether O of the ester (C15)
		np	hydroxyl O (C3) (3)	ester carbonyl O (C4)
		hb; np	amide N	ester carbonyl O (C4)
		hb; np	hydroxyl O (C3)	hydroxyl O (C3)
		np	glycosidic bond O 1-5 (2)	hydroxyl O (C3)
		hb; np	hydroxyl O (C6) (2)	hydroxyl O (C3)
		hb; np	hydroxyl O (C6) (2)	glycosidic bond O 2-11
134	99.0	np	hydroxyl O (C3) (1)	ester carbonyl O (C4)
		hb; np	amide N	ester carbonyl O (C4)
		hb; np	hydroxyl O (C3)	hydroxyl O (C3)
		np	glycosidic bond O 1-5 (2)	hydroxyl O (C3)
		hb; np	hydroxyl O (C6) (2)	hydroxyl O (C3)
		hb; np	hydroxyl O (C6) (2)	glycosidic bond O 2-11
		p	amine N (2)	ester carbonyl O (C15)
		hb	hydroxyl O (C6)	ester carbonyl O (C15)
		hb	glycosidic bond O 1-4 (2)	ester carbonyl O (C15)
		p	glycosidic bond O 1-4 (2)	ether O of the ester (C15)
135	99.1	hb	hydroxyl O (C6) (1)	ester carbonyl O (C15)

		p	amine N (1)	ester carbonyl O (C15)
		np	hydroxyl O (C6)	ester carbonyl O (C15)
		np	hydroxyl O (C6)	ether O of the ester (C15)
		p	glycosidic bond O 1-4 (2)	ether O of the ester (C15)
		hb; np	hydroxyl O (C3) (3)	ester carbonyl O (C4)
		hb; np	amide N	ester carbonyl O (C4)
		np	amide N	hydroxyl O (C3)
		np	hydroxyl O (C3)	ester carbonyl O (C4)
		hb; np	hydroxyl O (C3)	hydroxyl O (C3)
		np	glycosidic bond O 1-5 (2)	hydroxyl O (C3)
		np	glycosidic bond O 1-5 (2)	glycosidic bond O 2-11
		hb; np	hydroxyl O (C6) (2)	hydroxyl O (C3)
		hb; np	hydroxyl O (C6) (2)	glycosidic bond O 2-11
136	99.2	hb; np	N amide	ester carbonyl O (C4)
		np	hydroxyl O (C3)	ester carbonyl O (C4)
		hb; np	hydroxyl O (C3)	ether O of the ester (C4)
		hb; np	hydroxyl O (C3)	hydroxyl O (C3)
		hb; np	hydroxyl O (C6)	hydroxyl O (C3)
		hb; np	hydroxyl O (C6)	glycosidic bond O 2-11
		np	amine N	ester carbonyl O (C15)
		hb	glycosidic bond O 1-4	ester carbonyl O (C15)
		p	glycosidic bond O 1-4	ether O of the ester (C15)
137	99.3	np	hydroxyl O (C6) (1)	hepoxide O
		hb; np	hydroxyl O (C6) (1)	hydroxyl O (C3)
		p	glycosidic bond O 1-4 (1)	ether O of the ester (C15)
		hb	glycosidic bond O 1-4 (1)	ester carbonyl O (C15)
		hb	hydroxyl O (C3) (2)	ester carbonyl O (C15)
		p	amine N (2)	ester carbonyl O (C15)
		hb; np	amide N	ester carbonyl O (C4)
		np	hydroxyl O (C3)	ester carbonyl O (C4)
		np	hydroxyl O (C3)	hydroxyl O (C3)
		np	hydroxyl O (C3)	ether O of the ester (C15)
138	99.4	hb	hydroxyl O (C3) (1)	ester carbonyl O (C15)
		hb	hydroxyl O (C6)	ester carbonyl O (C15)

		p	glycosidic bond O 1-4 (2)	ether O of the ester (C15)
		hb; np	amide N	ester carbonyl O (C4)
		np	hydroxyl O (C3)	ester carbonyl O (C4)
		hb; np	hydroxyl O (C3)	hydroxyl O (C3)
		hb; np	hydroxyl O (C6) (2)	hydroxyl O (C3)
		hb; np	hydroxyl O (C6) (2)	glycosidic bond O 2-11
139	99.5	hb	hydroxyl O (C3) (1)	ester carbonyl O (C15)
		p	amine N (1)	ester carbonyl O (C15)
		hb	glycosidic bond O 1-4 (2)	ester carbonyl O (C15)
		p	glycosidic bond O 1-4 (2)	ether O of the ester (C15)
		np	hydroxyl O (C3) (2)	ester carbonyl O (C4)
		hb; np	amide N	ester carbonyl O (C4)
		hb; np	hydroxyl O (C3)	hydroxyl O (C3)
		np	glycosidic bond O 1-5 (2)	hydroxyl O (C3)
		hb; np	hydroxyl O (C6) (2)	hydroxyl O (C3)
		hb; np	hydroxyl O (C6) (2)	glycosidic bond O 2-11
140	99.6	hb	glycosidic bond O 1-4 (1-2)	ester carbonyl O (C15)
		np	hydroxyl O (C3) (1)	ester carbonyl O (C15)
		hb	glycosidic bond O 1-4 (3)	ester carbonyl O (C15)
		p	glycosidic bond O 1-4 (3)	ether O of the ester (C15)
		p	glycosidic bond O 1-5 (3)	ether O of the ester (C15)
		hb; np	hydroxyl O (C3)	ether O of the ester (C15)
		hb	amide carbonyl O	ether O of the ester (C4)
		hb; np	hydroxyl O (C3) (3)	hydroxyl O (C3)
141	99.7	np	hydroxyl O (C3)	ether O of the ester (C15)
		p	amide N	ether O of the ester (C15)
		np	amide carbonyl O	ester carbonyl O (C15)
142	99.8	np	hydroxyl O (C6) (1)	glycosidic bond O 2-11
		np	hydroxyl O (C6) (2)	ether O of the ester (C15)
		np	hydroxyl O (C6) (2)	ester carbonyl O (C15)
		hb	glycosidic bond O 1-5 (3)	ester carbonyl O (C15)
		hb	glycosidic bond O 1-4 (2)	ester carbonyl O (C15)
		np	hydroxyl O (C3)	ester carbonyl O (C15)
143	99.9	p	glycosidic bond O 1-5 (1)	hepoxide O

		np	hydroxyl O (C6) (2)	glycosidic bond O 2-11
		hb; np	hydroxyl O (C6) (3)	hydroxyl O (C3)
		np	hydroxyl O (C6) (3)	ether O of the ester (C15)
		hb	glycosidic bond O 1-5 (3)	ester carbonyl O (C15)
		hb	glycosidic bond O 1-4 (3)	ester carbonyl O (C15)
144	100	np	hydroxyl O (C6) (1)	hepoxide O
		np	hydroxyl O (C6) (1)	glycosidic bond O 2-11
		np	hydroxyl O (C6) (2)	hydroxyl O (C3)
		hb; np	hydroxyl O (C6) (2)	ester carbonyl O (C4)
		np	hydroxyl O (C6) (2)	ether O of the ester (C15)
		np	glycosidic bond O 1-5 (2)	ether O of the ester (C15)
		hb	glycosidic bond O 1-5 (2)	ester carbonyl O (C15)
		hb	glycosidic bond O 1-4 (2)	ester carbonyl O (C15)

Interaction types: hydrogen bond = hb; polar = p; non-polar = np. Numbers in parentheses indicate the number of involved D-glucosamine molecules in the interaction point in case there are more than one. All glycosidic bond 1-4 interactions were found between D-glucosamines.
