# Supplementary material for: Study of In Silico Binding Interactions and In Vitro Biosorption of Type A Trichothecenes Using Devil Fish Chitosan
Source: Toxins (Basel). 2026 Jun 10;18(6):263. doi: 10.3390/toxins18060263 (PMC13308271; doi:10.3390/toxins18060263)
Supplement: Supplementary file 1 [file toxins-18-00263-s001.zip › Table S4. chitosan-HT2 (HT2) binding poses.pdf]

Table S4. Contact points and interaction types between chitosan former molecules and the type A trichothecene HT2 toxin (HT2).

Chitosan-HT2 interactions					
Contact pose	Time (ns)	Interaction type	Binding Sites		
			Chitosan		HT2
			D-glucosamine	N-acetylglucosamine	
1	0.4	hb; np	amine N (1)		ester carbonyl O (C15)
2	14.0	np		amide carbonyl O	hydroxyl O (C4)
3	25.3	hb; np<br>hb; np<br>hb; np<br>np	hydroxyl O (C6) (1)<br>glycosidic bond O 1-4 (1-2)<br>amine N (1)<br>hydroxyl O (C6) (1)		hydroxyl O (C3)<br>hydroxyl O (C3)<br>hydroxyl O (C3)<br>glycosidic bond O 2-11
4	25.4	hb; np<br>hb; np<br>np	amine N (1)<br>amine N (1)<br>glycosidic bond O (1-4)		hydroxyl O (C3)<br>glycosidic bond O 2-11<br>hydroxyl O (C3)
5	40	hb		amide carbonyl O	ester carbonyl O (C15)
6	40.2	hb		amide carbonyl O	ester carbonyl O (C15)
7	60.2	hb; np<br>hb; np<br>np<br>np	hydroxyl O (C4) (1)<br>amine N (2)<br>hydroxyl O (C3) (2)<br>hydroxyl O (C4) (1)		ester carbonyl O (C15)<br>ester carbonyl O (C8)<br>ester carbonyl O (C8)<br>ether O of the ester (C15)
8	60.3	hb; np<br>np<br>hb; np	amine N (1)<br>hydroxyl O (C3) (1) 	 <br>hydroxyl O (C6)	hepoxide O<br>hepoxide O<br>hydroxyl O (C4)
9	60.6	hb; np	hydroxyl O (C6) (1)		glycosidic bond O 2-11
10	60.7	hb; np<br>np	hydroxyl O (C4) (1)<br>hydroxyl O (C6) (1)		glycosidic bond O 2-11<br>hydroxyl O (C3)
11	61.1	hb; np<br>np	amine N (1)<br>glycosidic bond O 1-4 (1-2)		hepoxide O<br>hepoxide O
12	61.2	p	glycosidic bond O 1-5 (1)		hepoxide O
13	61.4	np<br>np	glycosidic bond O 1-5 (1)<br>hydroxyl O (C3) (1)		hydroxyl O (C4)<br>hydroxyl O (C4)
14	61.5	np	glycosidic bond O 1-5 (1)		hepoxide O

15	61.6	np	glycosidic bond O 1-5 (1)		hydroxyl O (C4)
16	61.7	hb; np<br>np	amine N (1)<br>hydroxyl O (C3) (1)		hepoxide O<br>hepoxide O
17	62.4	hb; np	amine N (1)		ester carbonyl O (C15)
18	62.9	hb; np<br>p	hydroxyl O (C6) (1)<br>glycosidic bond O 1-5 (2)		hepoxide O<br>hepoxide O
19	63.2	hb; np<br>hb; np	hydroxyl O (C4) (1)<br>hydroxyl O (C4) (1)		hepoxide O<br>hydroxyl O (C4)
20	63.6	np<br>np<br>np<br>np	glycosidic bond O 1-5 (1)<br>glycosidic bond O 1-5 (2)<br>hydroxyl O (C3) (1)<br>hydroxyl O (C6) (2)		hepoxide O<br>hepoxide O<br>hepoxide O<br>hydroxyl O (C4)
21	63.7	hb; np<br>np	hydroxyl O (C6) (1)<br>glycosidic bond O 1-5 (1)		ester carbonyl O (C15)<br>hydroxyl O (C4)
22	63.9	hb; np	hydroxyl O (C6) (1)		hydroxyl O (C4)
23	64.1	np	hydroxyl O (C6) (1)		hydroxyl O (C4)
24	64.3	hb; np<br>np	hydroxyl O (C3) (1)<br>hydroxyl O (C4) (1)		hydroxyl O (C4)<br>hydroxyl O (C4)
25	64.4	hb; np<br>hb; np<br>np	hydroxyl O (C6) (1)<br>hydroxyl O (C3) (1)<br>hydroxyl O (C4) (2)		hydroxyl O (C4)<br>hydroxyl O (C4)<br>hydroxyl O (C4)
26	64.5	np<br>np<br>np	hydroxyl O (C6) (1)<br>hydroxyl O (C6) (1)<br>hydroxyl O (C4) (2)		hydroxyl O (C4)<br>hepoxide O<br>hepoxide O
27	64.6-<br>64.8	hb; np<br>hb; np	hydroxyl O (C3) (1)<br>hydroxyl O (C4) (1)		hydroxyl O (C4)<br>hydroxyl O (C4)
28	64.9	np<br>hb; np	hydroxyl O (C3) (1)<br>hydroxyl O (C4) (1)		hydroxyl O (C4)<br>hydroxyl O (C4)
29	65	hb; np<br>np<br>np	amine N (1)<br>hydroxyl O (C6) (2)<br>hydroxyl O (C4) (1)		hydroxyl O (C4)<br>hydroxyl O (C4)<br>ester carbonyl O (C15)
30	65.1	np<br>np	hydroxyl O (C6) (1)<br>hydroxyl O (C4) (2)		hydroxyl O (C4)<br>ester carbonyl O (C15)
31	65.2	hb; np	amine N (2)		hydroxyl O (C4)

		np<br>np	hydroxyl O (C6) (1)<br>hydroxyl O (C6) (2)		hydroxyl O (C4)<br>ester carbonyl O (C15)
32	65.3	np<br>np	hydroxyl O (C6) (1)<br>hydroxyl O (C4) (2)		hydroxyl O (C4)<br>ester carbonyl O (C15)
33	65.4	np<br>np<br>np<br>np	hydroxyl O (C4) (1)<br>hydroxyl O (C4) (1)<br>amine N (1)<br>hydroxyl O (C6) (2)		ester carbonyl O (C15)<br>ester carbonyl O (C15)<br>hydroxyl O (C4)<br>hydroxyl O (C4)
34	65.5	p<br>hb; np	glycosidic bond O 1-5 (1)<br>hydroxyl O (C3) (2)		hepoxide O<br>hepoxide O
35	65.6	hb; np<br>np<br>hb; np<br>np	hydroxyl O (C6) (1)<br>hydroxyl O (C6) (1)<br>amine N (2)<br>hydroxyl O (C3) (2)		hepoxide O<br>hydroxyl O (C4)<br>hydroxyl O (C4)<br>hydroxyl O (C4)
36	65.7	hb; np<br>hb; np	amine N (1)<br>hydroxyl O (C6) (2)		hydroxyl O (C4)<br>hydroxyl O (C4)
37	65.8	hb; np	amine N (1)		hydroxyl O (C4)
38	65.9-<br>66.0	np<br>np	amine N (1)<br>hydroxyl O (C6) (2)		hydroxyl O (C4)<br>hydroxyl O (C4)
39	66.2	np	hydroxyl O (C4) (1)		hydroxyl O (C4)
40	66.3	np<br>np<br>np	amine N (1)<br>hydroxyl O (C6) (2)<br>hydroxyl O (C6) (2)		hydroxyl O (C4)<br>hydroxyl O (C4)<br>hepoxide O
41	66.4	hb; np<br>np	amine N (1)<br>hydroxyl O (C6) (2)		hydroxyl O (C4)<br>hydroxyl O (C4)
42	66.5	hb; np	amine N (1)		hydroxyl O (C4)
43	66.6	hb; np<br>np	amine N (1)<br>hydroxyl O (C6) (2)		hydroxyl O (C4)<br>hydroxyl O (C4)
44	66.7-<br>66.8	hb; np	hydroxyl O (C6) (1)		hydroxyl O (C4)
45	66.9	np<br>np<br>np<br>np	hydroxyl O (C3) (1)<br>hydroxyl O (C6) (2)<br>hydroxyl O (C6) (3)<br>hydroxyl O (C4) (3)		hepoxide O<br>hydroxyl O (C4)<br>ester carbonyl O (C8)<br>ester carbonyl O (C15)

46	82.4	np	glycosidic bond O 1-4 (1)		ester carbonyl O (C15)
47	83.0	hb; np	amine N (1)		hydroxyl O (C3)

Interaction types: hydrogen bond = hb; polar = p; non-polar = np. Numbers in parentheses indicate the number of involved D-glucosamine molecules in the interaction point in case there are more than one. All glycosidic bond 1-4 interactions were found between D-glucosamines.
