# Supplementary material for: Study of In Silico Binding Interactions and In Vitro Biosorption of Type A Trichothecenes Using Devil Fish Chitosan
Source: Toxins (Basel). 2026 Jun 10;18(6):263. doi: 10.3390/toxins18060263 (PMC13308271; doi:10.3390/toxins18060263)
Supplement: Supplementary file 1 [file toxins-18-00263-s001.zip › Table S3. Chitosan-T2 toxin (T2) binding poses.pdf]

Table S3. Contact points and interaction types between chitosan former molecules and the type A trichothecene T2 toxin (T2).

Contact pose	Time (ns)	Interaction type	Binding Sites		
			Chitosan		T2
			D-glucosamine	N-acetylglucosamine	
1	0.1	hb; np	hydroxyl O (C6) (1)		ester carbonyl O (C15)
2	0.4	np	hydroxyl O (C6) (1)		2-11 glycosidic bond O
		np	hydroxyl O (C3) (2)		ester carbonyl O (C8)
3	0.5	np	amine N (1)		ester carbonyl O (C8)
4	0.6	hb; np	amine N (1)		ester carbonyl O (C8)
		np	hydroxyl O (C3) (1)		hepoxide O
		hb; np	hydroxyl O (C6) (2)		2-11 glycosidic bond O
5	0.9	hb; np	hydroxyl O (C6) (1)		ester carbonyl O (C8)
6	1	hb; np	hydroxyl O (C6) (1)		ester carbonyl O (C8)
		np	hydroxyl O (C3) (2)		ester carbonyl O (C8)
		np	hydroxyl O (C3) (2)		ether O of the ester (C8)
7	1.1	hb; np	hydroxyl O (C6) (1)		ester carbonyl O (C8)
		np	hydroxyl O (C3) (2)		ester carbonyl O (C8)
		np	hydroxyl O (C3) (2)		ether O of the ester (C8)
		hb; np	amine N (2)		ester carbonyl O (C15)
8	1.5	hb; np	hydroxyl O (C3) (1)		ester carbonyl O (C4)
9	1.6	np	hydroxyl O (C6) (1)		hepoxide O
10	1.7	hb; np	amine N (1)		ester carbonyl O (C15)
		np	hydroxyl O (C6) (2)		ester carbonyl O (C15)
		np	hydroxyl O (C6) (2)		ether O of the ester (C15)
		hb	glycosidic bond O 1-5 (2)		ester carbonyl O (C4)
		hb; np	hydroxyl O (C3)		ester carbonyl O (C4)
11	1.8	np	hydroxyl O (C6) (1)		ether O of the ester (C15)
		hb	glycosidic bond O 1-5 (1)		ester carbonyl O (C4)
		hb; np	hydroxyl O (C3) (2)		ester carbonyl O (C4)
		np	hydroxyl O (C3) (2)		hydroxyl O (C3)
		np	hydroxyl O (C3) (2)		ether O of the ester (C4)
		hb	amine N (2)		ether O of the ester (C4)
		np	amine N (2)		hydroxyl O (C3)

12	1.9	hb; np	hydroxyl O (C3) (1)		hydroxyl O (C3)
		hb; np	hydroxyl O (C3) (1)		2-11 glycosidic bond O
		hb; np	amine N (1)		2-11 glycosidic bond O
13	2.0	hb	glycosidic bond O 1-5 (1)		ester carbonyl O (C4)
		hb	glycosidic bond O 1-4 (1-2)		ester carbonyl O (C4)
		hb	amine N (3)		ester carbonyl O (C4)
		hb	hydroxyl O (C3) (3)		ester carbonyl O (C4)
		np	hydroxyl O (C3) (3)		hydroxyl O (C3)
		np	amine N (3)		hydroxyl O (C3)
14	2.1	hb; np	hydroxyl O (C6) (1)		ester carbonyl O (C15)
		hb; np	amine N (2)		hydroxyl O (C3)
		np	hydroxyl O (C3) (2)		ester carbonyl O (C4)
		np	amine N (3)		hydroxyl O (C3)
		np	hydroxyl O (C3) (3)		hydroxyl O (C3)
		np	hydroxyl O (C3) (3)		ester carbonyl O (C4)
15	2.2	hb; np	amine N (1)		ether O of the ester (C15)
		np	hydroxyl O (C3) (1)		ester carbonyl O (C15)
		np	hydroxyl O (C3) (1)		ether O of the ester (C15)
		hb; np	amine N (2)		hydroxyl O (C3)
		np	hydroxyl O (C3) (2)		hydroxyl O (C3)
		np	hydroxyl O (C3) (2)		ester carbonyl O (C4)
		hb; np	hydroxyl O (C6) (3)		ester carbonyl O (C4)
		hb	glycosidic bond O 1-5 (3)		ester carbonyl O (C4)
16	2.3	hb; np	hydroxyl O (C3) (1)		ester carbonyl O (C4)
		hb; np	amine N (1)		hydroxyl O (C3)
17	2.4	hb; np	hydroxyl O (C3) (1)		ester carbonyl O (C15)
		np	hydroxyl O (C6) (2)		hydroxyl O (C3)
		np	hydroxyl O (C6) (2)		ester carbonyl O (C4)
18	2.5	hb	glycosidic bond O 1-4		ester carbonyl O (C4)
19	2.6	np	hydroxyl O (C6) (1)		ester carbonyl O (C4)
20	2.8	np		amide carbonyl O	hydroxyl O (C3)
		np	hydroxyl O (C3) (1)		ester carbonyl O (C4)
21	2.9	hb; np	hydroxyl O (C3) (1)		hydroxyl O (C3)
		np	glycosidic bond O 1-4		hydroxyl O (C3)

		np		glycosidic bond O 1-4		glycosidic bond O 2-11
		np			amide carbonyl O	hepoxide O
22	27.1	np	hydroxyl O (C6) (1)			hepoxide O
23	51.4	hb			amide carbonyl O	hydroxyl O (C3)
24	52.8	hb			amide carbonyl O	ester carbonyl O (C15)
25	52.9	hb; np			hydroxyl O (C3)	hydroxyl O (C3)
		np		glycosidic bond O 1-4		hydroxyl O (C3)
		np	glycosidic bond O 1-5 (1)			hydroxyl O (C3)
		np	hydroxyl O (C6) (1)			hydroxyl O (C3)
		np	hydroxyl O (C6) (1)			ester carbonyl O (C4)
26	53	np		glycosidic bond O 1-4		hydroxyl O (C3)
		np	glycosidic bond O 1-5 (1)			ether O of the ester (C15)
		np	hydroxyl O (C6) (1)			ester carbonyl O (C4)
27	53.1	hb; np			hydroxyl O (C3)	hydroxyl O (C3)
		np			hydroxyl O (C3)	hydroxyl O (C3)
		hb; np		glycosidic bond O 1-4		ester carbonyl O (C4)
		np	glycosidic bond O 1-5 (1)			ester carbonyl O (C4)
		np			amide N	ester carbonyl O (C4)
28	53.2	np			amide N	hydroxyl O (C3)
		np			hydroxyl O (C3)	hydroxyl O (C3)
		hb; np			hydroxyl O (C3)	ester carbonyl O (C4)
		hb; np	hydroxyl O (C6)			ester carbonyl O (C4)
		np	glycosidic bond O 1-5			ester carbonyl O (C4)
29	53.3	hb; np			amide N	hydroxyl O (C3)
		np			hydroxyl O (C3)	hydroxyl O (C3)
30	66.1-	hb			acetyl carbonyl O	ether O of the ester (C4)
	66.2	np		glycosidic bond O 1-4		ether O of the ester (C4)
		np		glycosidic bond O 1-4		hepoxide O
		p			glycosidic bond O 1-5	hepoxide O
		hb; np	hydroxyl O (C3) (1)			hepoxide O
		hb; np			hydroxyl O (C6)	hepoxide O
31	66.7	hb; np	hydroxyl O (C3) (1)			hepoxide O
		p	glycosidic bond O 1-4 (1-2)			hepoxide O
		p	glycosidic bond O 1-5 (2)			hepoxide O

32	71.7	hb; np	hydroxyl O (C1) (1)		ester carbonyl O (C8)
33	71.9	hb; np	hydroxyl O (C6) (1)		ester carbonyl O (C15)
34	84.9	np	hydroxyl O (C6) (1)		ether O of the ester (C8)
35	85.1	np<br>np	glycosidic bond O 1-5 (1)<br>hydroxyl O (C3) (2)		ester carbonyl O (C8)<br>hepoxide O
36	85.2	np<br>np	hydroxyl O (C6) (1)<br>glycosidic bond O 1-5 (2)		ester carbonyl O (C8)<br>ester carbonyl O (C8)
37	85.3	hb; np<br>p<br>np<br>hb; np<br>hb; np	hydroxyl O (C6) (1)<br>glycosidic bond O 1-5 (2)<br><br><br>hydroxyl O (C6) (2)	 <br>hydroxyl O (C3)<br>amide N 	ether O of the ester (C4)<br>hepoxide O<br>hepoxide O<br>ester carbonyl O (C8)<br>ester carbonyl O (C8)
38	85.4	np<br>np<br>np<br>np	hydroxyl O (C6) (1)<br>hydroxyl O (C6) (1)<br>hydroxyl O (C3) (2) 	 <br><br>hydroxyl O (C3)	ester carbonyl O (C4)<br>ether O of the ester (C4)<br>ester carbonyl O (C8)<br>ester carbonyl O (C8)
39	85.5	np<br>np<br>hb; np	glycosidic bond O 1-4 (1-2)<br>hydroxyl O (C3) (1)<br>amine N (1)		ester carbonyl O (C8)<br>hepoxide O<br>hepoxide O
40	98.9	hb		amide carbonyl O	ester carbonyl O (C15)
41	99.0-99.1	hb		amide carbonyl O	ester carbonyl O (C4)
42	99.2	hb; np		amide carbonyl O	ester carbonyl O (C4)
43	99.3	hb; np<br>hb		amide carbonyl O<br>amide carbonyl O	ester carbonyl O (C15)<br>ester carbonyl O (C4)
44	99.4	np<br>np		amide carbonyl O<br>amide carbonyl O	ester carbonyl O (C4)<br>ether O of the ester (C4)
45	99.5	hb<br>np<br>np<br>hb		amide carbonyl O<br>amide carbonyl O<br>amide carbonyl O<br>amide carbonyl O	ester carbonyl O (C15)<br>ether O of the ester (C15)<br>ether O of the ester (C4)<br>ester carbonyl O (C4)
46	99.6	np		amide carbonyl O	ester carbonyl O (C4)
47	99.7	hb; np<br>hb	hydroxyl O (C6) (1) 	 amide carbonyl O	ester carbonyl O (C15)<br>ester carbonyl O (C4)

48	99.8	hb; np	hydroxyl O (C3) (1)		ester carbonyl O (C15)
49	99.9	hb; np<br>np<br>np	amine N (1)<br>hydroxyl O (C3) (1)<br>hydroxyl O (C3) (1)		hepoxide O<br>hepoxide O<br>glycosidic bond O 2-11
50	100	np	hydroxyl O (C6)		hydroxyl O (C3)

Interaction types: hydrogen bond = hb; polar = p; non-polar = np. Glycosidic bond O 1-4 interactions in the D-glucosamine column indicate the bond between two D-glucosamines. While Glycosidic bond O 1-4 centered at the middle of the D-glucosamine and N-acetylglucosamine columns indicate that the bond is between a D-glucosamine and an N-acetylglucosamine molecules.
