# Supplementary material for: Study of In Silico Binding Interactions and In Vitro Biosorption of Type A Trichothecenes Using Devil Fish Chitosan
Source: Toxins (Basel). 2026 Jun 10;18(6):263. doi: 10.3390/toxins18060263 (PMC13308271; doi:10.3390/toxins18060263)
Supplement: Supplementary file 1 [file toxins-18-00263-s001.zip › Table S2. Chitosan-neosolaniol (NEO) binding poses.pdf]

Table S2. Contact poses and interaction types between chitosan former molecules and the type A trichothecene neosolaniol (NEO).

Contact pose	Time (ns)	Interaction type	Binding Sites		
			Chitosan		NEO
			D-glucosamine	N-acetylglucosamine	
1	0.2	hb; np	amine N (1)		ester carbonyl O (C4)
		np	hydroxyl O (C3) (1)		ester carbonyl O (C4)
		np	hydroxyl O (C3) (1)		hydroxyl O (C3)
		np	glycosidic bond O 1-5 (2)		hydroxyl O (C3)
		hb; np	hydroxyl O (C6) (2)		hydroxyl O (C3)
		hb; np	hydroxyl O (C6) (2)		glycosidic bond O 2-11
2	0.3	np	amine N (1)		hydroxyl O (C3)
		np	hydroxyl O (C3) (1)		ester carbonyl O (C4)
		np	hydroxyl O (C3) (1)		ether O of the ester (C4)
		np	hydroxyl O (C3) (1)		hydroxyl O (C3)
		hb; np	hydroxyl O (C6) (2)		hydroxyl O (C3)
3	0.4	np	hydroxyl O (C6) (1)		hydroxyl O (C3)
4	0.6	hb		amide-carbonyl O	carbonyl O of the ester (C15)
5	0.7	hb; np	glycosidic bond O 1-5 (1)		hydroxyl O (C8)
6	0.9	np	glycosidic bond O 1-5 (1)		hydroxyl O (C3)
		np	glyosidic bond O 1-5 (2)		hydroxyl O (C3)
7	1	np	glycosidic bond O 1-5 (1)		hydroxyl O (C3)
		hb		amide-carbonyl O	ether O of the ester (C15)
		np		amide-carbonyl O	ester carbonyl O (C15)
8	1.1	np	hydroxyl O (C6) (1)		ester carbonyl O (C4)
		hb; np	hydroxyl O (C3) (2)		ester carbonyl O (C15)
		hb; np	glycosidic bond O 1-5 (1)		ester carbonyl O (C15)
9	1.2	hb; np	amine N (1)		ester carbonyl O (C4)
		np	hydroxyl O (C3) (1)		ester carbonyl O (C15)
		np	hydroxyl O (C3) (1)		ether O of the ester (C15)
10	27.8	hb; np	hydroxyl O (C6) (1)		ester carbonyl O (C15)
11	28.3	np	hydroxyl O (C4) (1)		hepoxide O
12	28.4	hb; np	hydroxyl O (C1) (1)		hepoxide O
13	28.6	hb	glycosidic bond O 1-5 (1)		ester carbonyl O (C4)

		np<br>np	hydroxyl O (C1) (1)<br>hydroxyl O (C1) (1)		ester carbonyl O (C4)<br>hydroxyl O (C3)
14	45.1	hb; np<br>np<br>hb	hydroxyl O (C3) (1)<br>hydroxyl O (C3) (1)<br>amine N (1)		hydroxyl O (C8)<br>ester carbonyl O (C15)<br>ester carbonyl O (C15)
15	45.2	hb; np	amine N (1)		hydroxyl O (C8)
16	45.3	np<br>hb; np	hydroxyl O (C3) (1)<br>amine N (1)		hydroxyl O (C8)<br>hydroxyl O (C8)
17	45.5	np<br>hb; np	hydroxyl O (C6) (1)<br>hydroxyl O (C6) (1)		ether O of the ester (C15)<br>hydroxyl O (C8)
18	47.6	np<br>hb; np<br>np<br>hb; np	hydroxyl O (C6) (1)<br>hydroxyl O (C4) (1)<br>hydroxyl O (C3) (1)<br>hydroxyl O (C3) (1)		ester carbonyl O (C15)<br>ester carbonyl O (C15)<br>hydroxyl O (C3)<br>ester carbonyl O (C4)
19	47.9	hb; np<br>np<br>hb<br>hb<br>hb; np<br>hb; np<br>np	amine N (1)<br>hydroxyl O (C3) (1)<br>glycosidic bond O 1-4 (1-2)<br>glycosidic bond O 1-5 (1)<br>hydroxyl O (C3) (2)<br>hydroxyl O (C6) (1)<br>hydroxyl O (C6) (1)		ester carbonyl O (C15)<br>ester carbonyl O (C15)<br>ester carbonyl O (C4)<br>ester carbonyl O (C4)<br>ester carbonyl O (C4)<br>ester carbonyl O (C4)<br>hydroxyl O (C3)
20	48	np<br>np<br>hb; np<br>np<br>np	hydroxyl O (C6) (1)<br>glycosidic bond O 1-5 (1)<br>glycosidic bond O 1-4 (1-2)<br>hydroxyl O (C3) (2)<br>hydroxyl O (C3) (2)		hydroxyl O (C3)<br>hydroxyl O (C3)<br>hydroxyl O (C3)<br>hydroxyl O (C3)<br>ester carbonyl O (C4)
21	48.1	np<br>hb; np<br>hb; np	hydroxyl O (C6) (1)<br>hydroxyl O (C4) (1)<br>hydroxyl O (C4) (1)		hydroxyl O (C3)<br>hydroxyl O (C3)<br>2-11 glycosidic bond O
22	52.5	hb; np	hydroxyl O (C4) (1)		ester carbonyl O (C4)
23	58.2	p<br>hb; np	glycosidic bond O 1-5 (1)<br>hydroxyl O (C1) (1)		hepoxide O<br>hepoxide O
24	58.3	hb; np	hydroxyl O (C6) (1)		hydroxyl O (C3)
25	58.4	np	hydroxyl O (C6) (1)		ester carbonyl O (C4)

26	76.1	np<br>hb; np	hydroxyl O (C3) (1)<br>amine N (1)		hydroxyl O (C8)<br>hydroxyl O (C8)

Interaction types: hydrogen bond = hb; polar = p; non-polar = np. Numbers in parentheses indicate the number of involved D-glucosamine molecules in the interaction point in case there are more than one. All glycosidic bond 1-4 interactions were found between D-glucosamines.
